# Revealing Missing Parts of the Interactome via Link Prediction

**DOI:** 10.1371/journal.pone.0090073

**Published:** 2014-03-03

**Authors:** Yuriy Hulovatyy, Ryan W. Solava, Tijana Milenković

**Affiliations:** Department of Computer Science and Engineering, ECK Institute for Global Health, and Interdisciplinary Center for Network Science and Applications, University of Notre Dame, Notre Dame, Indiana, United States of America; Semmelweis University, Hungary

## Abstract

Protein interaction networks (PINs) are often used to “learn” new biological function from their topology. Since current PINs are noisy, their computational de-noising via link prediction (LP) could improve the learning accuracy. LP uses the existing PIN topology to predict missing and spurious links. Many of existing LP methods rely on shared *immediate* neighborhoods of the nodes to be linked. As such, they have limitations. Thus, in order to *comprehensively* study what are the topological properties of nodes in PINs that dictate whether the nodes should be linked, we introduce novel *sensitive* LP measures that are expected to overcome the limitations of the existing methods.

We systematically evaluate the new and existing LP measures by introducing “synthetic” noise into PINs and measuring how accurate the measures are in reconstructing the original PINs. Also, we use the LP measures to de-noise the original PINs, and we measure biological correctness of the de-noised PINs with respect to functional enrichment of the predicted interactions. Our main findings are: 1) LP measures that favor nodes which are *both* “topologically similar” *and* have large shared *extended* neighborhoods are superior; 2) using more network topology often though not always improves LP accuracy; and 3) LP improves biological correctness of the PINs, plus we validate a significant portion of the predicted interactions in independent, external PIN data sources.

Ultimately, we are less focused on identifying a superior method but more on showing that LP improves biological correctness of PINs, which is its ultimate goal in computational biology. But we note that our new methods outperform each of the existing ones with respect to at least one evaluation criterion. Alarmingly, we find that the different criteria often disagree in identifying the best method(s), which has important implications for LP communities in any domain, including social networks.

## Introduction

### Motivation and background

Networks (or graphs) model real-world phenomena in many domains. We focus on biological networks, protein-protein interaction (PPI) networks in particular, with the goal of identifying missing and spurious links in current noisy PPI networks. Nonetheless, our study is applicable to other network types as well. In PPI networks, nodes are proteins and two nodes are connected by an edge if the corresponding proteins interact in the cell. We focus on these networks, since it is the proteins (gene products) that carry out the majority of cellular processes and they do so by interacting with other proteins. And this is exactly what PPI networks model.

High-throughput methods for PPI detection, e.g., yeast two-hybrid (Y2H) assays or affinity purification followed by mass spectrometry (AP/MS), have produced PPI data for many species [1]–[5]. However, current networks are *noisy*, with many missing and spurious PPIs, due to limitations of biotechnologies as well as human biases [6]–[10]. AP/MS is estimated to have a 15–50% false positive rate and a 63–77% false negative rate [11]. Similar holds for Y2H, though PPIs obtained by Y2H are still more precise than literature-curated PPIs supported by a single publication [12].

Analogous to genomic sequence research, biological network research is promising to revolutionize our biological understanding: prediction of protein function and the role of proteins in disease from PPI network topology has already received attention [13]–[17]. However, the noisiness of the network data is an obstacle on this promising avenue, as it could lead to incorrect predictions. Computational de-noising of current PPI network data by identifying missing and spurious links could improve the quality of topology-based predictions and consequently save resources needed for experimental validation of the predictions. Thus, we aim to test how well we can decrease the noise in PPI data via link prediction (LP).

LP typically uses the existing topology of the network to predict missing and spurious links [18]–[24]. Alternatively, one network type, e.g., functional interactions, can be used to predict another network type, e.g., physical PPIs [24]. LP consists of unsupervised or supervised approaches that use some measure of the topology of the nodes to be linked [21]. For example, it may be desirable to link nodes with high degrees as measured by preferential attachment [25]–[28], nodes that share many neighbors as measured by Jaccard [29] or Adamic/Adar coefficients [30], nodes that share many paths as measured by Katz index [31], or similar [32]. Both supervised and unsupervised LP methods have their (dis)advantages. Though supervised methods can outperform unsupervised ones, much of previous research has focused on unsupervised LP, since many factors that might influence supervised LP have not been well understood [18], [21], [22].

There are some limitations to the existing LP measures. With some exceptions [31], [33], most of them capture only the topological information contained in the *immediate* network neighborhood of nodes to be linked [25], [26], [29], [30], [34]. However, significant amount of the information is available in the rest of the network that could improve LP accuracy. Thus, additional sensitive measures that capture deeper network topology might be needed. We recently generalized the idea of shared immediate neighborhoods to shared *extended* neighborhoods in the context of network clustering and showed that including more network topology resulted in biologically superior clusters [35]. So, it is reasonable to test whether including more topology will be effective for LP as well.

Also, most of the existing shared neighborhood-based methods can predict a link only between nodes that are within the shortest path *distance of two* from each other [29], [30], [34], whereas it might be beneficial to link nodes which are *more distant* [31], [33]. Preferential attachment-based measures can achieve this [25], [26], but they again capture only the immediate neighborhoods of the nodes to be linked. A shortest path-based LP method exists which can also connect distant nodes in the network but which can at the same time capture deeper network topology. However, this method is computationally expensive [36]. A couple of additional methods exist that can link distant nodes under the hypothesis that nodes that share many paths or that are at similar distances to all other nodes in the network should be linked [31], [33]. Here, we introduce an alternative and sensitive measure of the topological similarity of *extended* neighborhoods of two nodes that addresses all of the above issues, and we use it with a *novel hypothesis* that nodes that are topologically similar should be linked together.

Another drawback of the existing methods is as follows. It might be more efficient to predict the existence of a link between two nodes by explicitly measuring the topological position of an *edge* (or equivalently a non-edge) rather than by measuring the position of each of the two *nodes* individually [37], as most of the current methods do. Thus, we propose a new, sensitive measure of the network position of an *edge* and a *non-edge*, which counts the number of subgraphs that the two nodes in question participate in *simultaneously*, and we use it with the hypothesis that nodes that participate in many subgraphs and thus have large and dense *extended* shared neighborhoods should be linked together.

### Our approach

We study several PPI networks of yeast, the best studied species to date, obtained by different experimental methods for PPI detection, and we apply our new as well as popular existing LP measures to the networks to de-noise them. Given a network, we aim to study the topologies of each node pair in it with respect to the given LP measure, in order to determine which of the node pairs should be connected.

We perform three types of evaluation tests, as follows. **First,** we introduce synthetic noise in the given PPI network by randomly removing a percentage of edges from the network, with the goal of measuring how well the given method can reconstruct the original network, using the original PPIs as the ground truth data. **Second,** given the availability of low-confidence PPI data for one of the studied networks, we apply the given method to this network and use the corresponding low-confidence PPI data as the ground truth data when evaluating the method. In both of these evaluation tests, we test the accuracy of a LP method in systematic receiver-operator curve and precision-recall settings. In this context, we study the effects on LP accuracy of the “topological similarity” as well as the size of the shared *extended* neighborhoods of nodes, where the nodes *can* be distant in the network. Also, we study what amount of network topology should be used for LP. We find that LP measures that favor nodes which are *both* topologically similar and which have large shared extended neighborhoods are superior to LP measures that have only one of these two properties. Also, we show that using more network topology often though not always increases LP accuracy.

**Third,** we apply the LP methods to the original PPI networks to de-noise them, and we evaluate the quality of the de-noised networks, i.e., of different LP methods, in two ways. First, we compute their biological correctness by measuring the “enrichment” of predicted edges in Gene Ontology (GO) terms [38]. Importantly, we show that LP improves the biological correctness of the PPI networks by de-noising them. Second, we search for the predicted interactions in an external, independent PPI data source, and in this way, we validate a significantly large portion of the predictions, further confirming the biological correctness of the de-noised networks.

Importantly, we show that our new LP measures are statistically significantly superior to each of the existing ones with respect to at least one of the evaluation criteria. Alarmingly, we find that receiver-operator curve, precision-recall, and biological (functional) evaluation frameworks do not necessarily agree in identifying the best LP method(s), which has important implications for the LP community.

## Methods

We study multiple *S. cerevisiae* PPI networks obtained by different experimental methods for PPI detection. Given a network, we aim to de-noise the network, with the goal of determining which of all pairs of nodes in the network should be connected by edges, with respect to a variety of existing as well as new LP measures. We evaluate the different measures in systematic precision-recall and receiver-operator curve frameworks, as well as with respect to two biological (functional) criteria. The details are as follows.

### Network data

We evaluate all LP methods on three *S. cerevisiae* yeast PPI networks obtained with different experimental methods. We study PPI networks of *yeast* because yeast has been the most studied species to date. As such, it has the most complete interactome and thus represents the best species to evaluate the methods on. We study *multiple* yeast PPI networks obtained with *different* experimental methods for PPI detection to test whether LP results are dependent on the experimental method. The three networks are: 1) *Y2H* network, obtained by Y2H, which consists of 1,647 nodes and 2,518 edges [3], [35]; 2) *AP/MS* network, obtained by AP/MS, which consists of 1,004 nodes and 8,319 edges [3], [35]; and 3) *high-confidence (HC)* network, obtained from multiple data sources, which consists of 1,004 nodes and 8,323 edges [9]. The quality of PPIs in the HC network is comparable to the quality of interactions produced by precise small-scale biological experiments [9]. Importantly, in addition to the high-confidence PPIs, the data by [9] also contains the corresponding lower-confidence PPI data, which is useful for evaluation of the LP methods (as explained below).

### Existing commonly used LP measures

#### Degree-based measure

According to preferential attachment [25], [26], the higher the degrees of two nodes, the more likely the nodes are to interact. The *degree product (DP)* measure scores the potential edge between two nodes *v* and *w* as: DP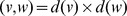, where 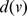 is the degree of node *v* [21].

#### Common neighbors-based measures

A popular idea is that the more neighbors (or paths) two nodes share, the more likely the nodes are to interact. Hence, a number of methods has been proposed in this context, as follows.

The *shared neighbors (SN)* measure scores the potential edge between nodes *v* and *w* as: SN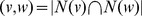, where 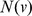 is the set of neighbors of *v* [25], [26]. SN simply counts the shared neighbors.

*Jaccard coefficient (JC)* scores the potential edge between two nodes *v* and *w* as: JC
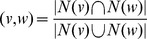
 [29]. That is, it scores two nodes with respect to the size of their shared neighborhood relative to the size of their entire neighborhoods combined. As such, it favors node pairs for which a high percentage of all neighbors are shared.

The *Adamic-Adar (AA)* measure scores the potential edge between two nodes *v* and *w* as: AA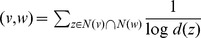 [30]. Thus, of all common neighbors of two nodes, it favors low-degree shared neighbors over high-degree shared neighbors.

*Katz index (Katz)* scores the potential edge between two nodes *v* and *w* as as follows: 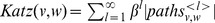, where 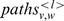 is the set of all paths between *v* and *w* having length of exactly *l*, and 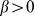 is a parameter that controls relative weights (i.e., levels of importance) of paths of different lengths [31], such that the smaller the value of *β*, the smaller the contribution of larger paths is to the sum. For our evaluation, we use a popular choice of value for *β* of 0.005 [20]. In summary, Katz favors node pairs that share many paths of different lengths.

*Local path index (LPI)* scores the potential edge between two nodes *v* and *w* as follows: 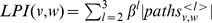 [39]. By considering paths of length 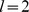 and 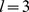, LPI provides a trade-off between SN (which considers only 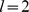) and Katz (which considers all possible values of *l*).

*Resource allocation index (RAI)* scores the potential edge between two nodes *v* and *w* as: 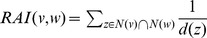 [34]. This measure, motivated by the resource allocation process taking place on networks, is similar to AA, the only difference being scaling of the denominator. For networks with small average degree, the results of AA and RAI are expected to be similar [34].

*Random walk with resistance (RWS)* scores the potential edge between two nodes *v* and *w* under the intuition that nodes having similar “distances” to all other nodes in the network are likely to interact with each other [33]; here, the distance is defined as the expected number of steps needed for a random walker to travel between two nodes in question. As such, RWS can predict links between nodes that are not necessarily close to each other and thus might not share any common neighbors. For a formal description, see the original publication [33].

### New LP measures

We already designed sensitive measures of topology that unlike many of the existing measures go beyond capturing only the direct neighborhoods of nodes to be linked. We used them for network alignment [40]–[42], clustering [35], [43]–[45], and modeling [46], [47], but they have not been used for LP thus far. Thus, we introduce them as new LP measures. Also, we design conceptually new measures. (Software executables are freely available upon request.) The details are as follows.

### Existing sensitive measures of topology as new LP measures

To go beyond capturing only the direct network neighborhood of a node, we previously designed a constraining graphlet-based measure of topology, called *node graphlet degree vector (node-GDV)*, that captures up to 4-deep neighborhood of a node; a graphlet is a small induced subgraph of the network [48]. We designed a measure of topological similarity of such extended neighborhoods of two nodes, called *node-GDV-similarity*. In this study, we use node-GDV-similarity for LP, with the hypothesis that the more topologically similar two nodes are, the more likely the nodes are to interact. Also, since shared neighbors-based approaches, which are among the best LP measures over the widest range of real-world networks [21], are based on the number of 3-node paths that two nodes in question share, where a 3-node path is just a 3-node graphlet, we generalize these measures by counting the number of all 3–5-node graphlets that the two nodes share. We do this by using a sensitive measure called *edge-GDV*. The formal description of all of the measures is as follows.

#### Node graphlet degree vector (node-GDV)

**Figure 1:**
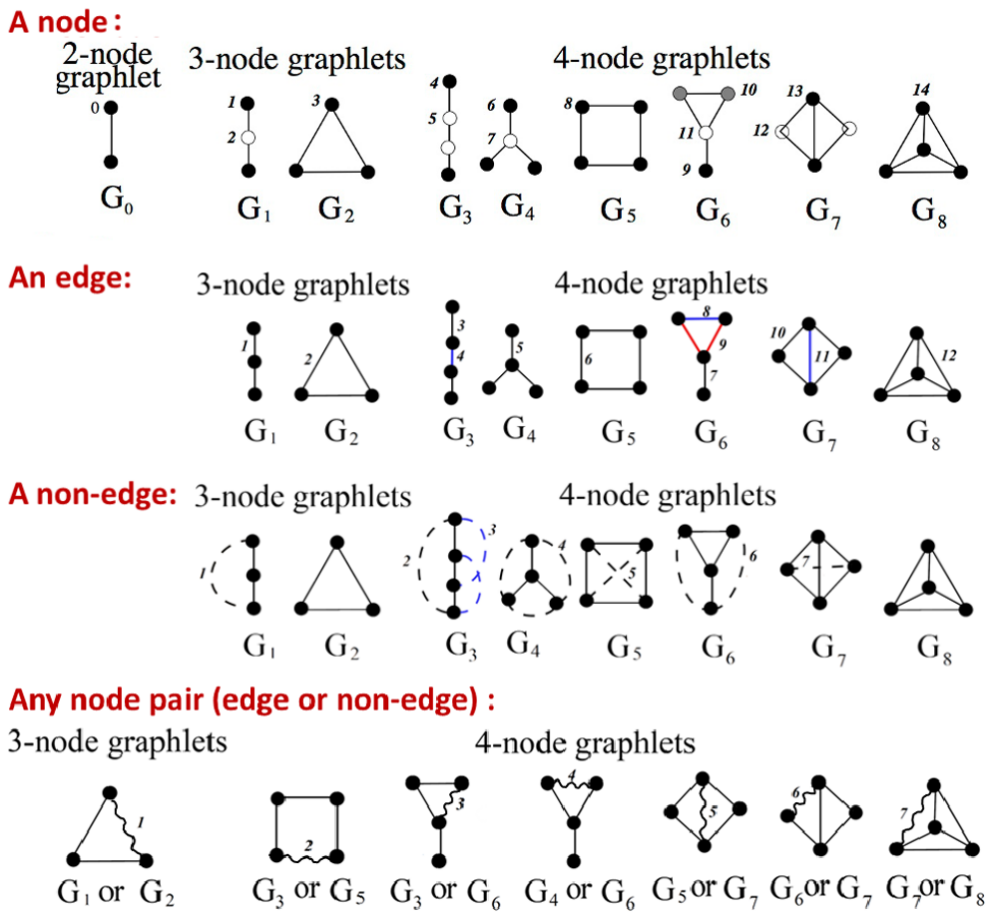
Graphlet positions of a node, an edge, a non-edge, and a node pair. All topological positions (“orbits”) in up to 4-node graphlets of a node (top; node shade), an edge (upper middle; solid line), a non-edge (lower middle; broken line), and any node pair, an edge or a non-edge (bottom; wavy line) are shown. For example: 1) in graphlet 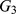, the two end nodes are in node orbit 4, while the two middle nodes are in node orbit 5; 2) in 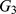, the two “outer” edges are in edge orbit 3, while the “middle” edge is in edge orbit 4; 3) in 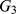, the non-edge touching the end nodes is in non-edge orbit 2, while the two non-edges that touch the end nodes and the middle nodes are in non-edge orbit 3; 4) a node pair at node pair orbit 1 touches a 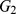 at edge orbit 2, if it is an edge, or a 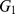 at non-edge orbit 1, if it is a non-edge (hence, mutually exclusive edge orbit 2 and non-edge orbit 1 are reconciled into a common node pair orbit 1). There are 15 node, 12 edge, 7 non-edge, and 7 node pair orbits for up to 4-node graphlets. In a graphlet, different orbits are colored differently. All up to 5-node graphlets are used, but only up to 4-node graphlets are illustrated. There are 73 node, 68 edge, 49 non-edge, and 49 node pair orbits for up to 5-node graphlets.

We generalized the degree of node *v* that counts the number of edges that *v* touches (where an edge is the only 2-node graphlet, denoted by 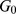 in Fig. 1), into *node-GDV* of *v* that counts the number of 2–5-node graphlets that *v* touches [43]. We need to distinguish between *v* touching, for example, a three-node path (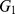 in Fig. 1) at an end node or at the middle node, because the end nodes are topologically identical to each other, while the middle node is not. This is because an automorphism (defined below) of 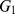 maps the end nodes to one another and the middle node to itself. Formally, an isomorphism *f* from graph *X* to graph *Y* is a bijection of nodes of *X* to nodes of *Y* such that *xy* is an edge of *X* if and only if 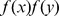 is an edge of *Y*. An automorphism is an isomorphism from X to itself. The automorphisms of *X* form the automorphism group, 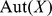. If *x* is a node of *X*, then the automorphism node orbit of *x* is 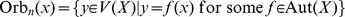, where 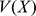 is the set of nodes of *X*. There are 73 node orbits for 2–5-node graphlets. Hence, node-GDV of *v* has 73 elements counting how many node orbits of each type touch *v* (*v*'s degree is the first element). It captures *v*'s up to 4-deep neighborhood and thus a large portion of real networks, as they are small-world [49].

#### Node-GDV-similarity

To compare node-GDVs of two nodes, one could use some existing measure, e.g., Euclidean distance. However, this might be inappropriate, as some orbit counts are not independent. Hence, we designed a new measure, called node-GDV-similarity, as follows [43]. For a node 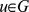, 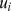 is the 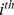 element of its node-GDV. The distance between the 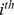 orbits of nodes *u* and *v* is 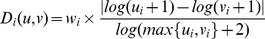, where 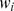 is the weight of orbit *i* that accounts for orbit dependencies [43]. The *log* is used because the 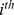 elements of two node-GDVs can differ by several orders of magnitude and we did not want the distance between node-GDVs to be dominated by large values. The total distance is 
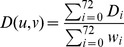
. Finally, node-GDV-similarity is 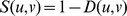. The higher the node-GDV-similarity between nodes, the higher their topological similarity.

#### Edge-GDV

Since a graphlet contains both nodes *and* edges, we defined *edge-GDV* to count the number of graphlets that an *edge* touches at a given “edge orbit” (Fig. 1) [35]. Given the automorphism group of graph *X*, 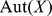, if 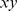 is an edge of 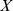, the edge orbit of 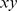 is 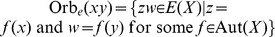, where 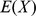 is the set of edges of 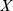. There are 68 edge orbits for 3–5-node graphlets [35]. (We designed *edge-GDV-similarity* to measure topological similarity of *edges*, which we used for network clustering [35]. However, we do not use this measure for LP.)

### Conceptually novel measures of topology

We need to predict the existence of a link between nodes independent on whether there is an edge between them in the original network. Thus, in addition to describing the network position of an edge, we need to be able to describe the position of a non-edge as well. Hence, we generalize edge-GDV into *non-edge-GDV* to measure the topological position of a non-edge. Then, we reconcile mutually exclusive edge-GDVs and non-edge-GDVs into a new *node-pair-GDV* measure, which counts the number of graphlets that a node pair (an edge or a non-edge) touches at a given “node pair orbit” (defined below). Finally, based on node-pair-edge-GDV of a node pair, we create a new measure of the topological centrality of the node pair, called *node-pair-GDV-centrality*. According to this measure, the more graphlets the two nodes participate in (or share), the higher their centrality. Then, node-pair-GDV-centrality is used as a LP measure to score potential edges between node pairs in the network. The measures are defined as follows.

#### Non-edge-GDV

Analogous to edge-GDV, in this study, we define *non-edge-GDV* to count the number of graphlets that a *non-edge* touches at a given “non-edge orbit” (Fig. 1). We define non-edge orbits as follows. If *xy* is a non-edge of graph *X*, then the non-edge orbit of *xy* is 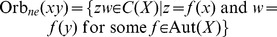, where 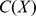 is the set of all non-edges of *X*. For example, in Fig. 1, in graphlet 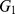, the only non-edge is in non-edge orbit 1. Graphlet 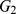 has no non-edges. In graphlet 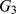, the non-edge that touches the two end nodes is in one non-edge orbit (non-edge orbit 2), while the remaining two non-edges that touch the end nodes and the middle nodes are in a different non-edge orbit (non-edge orbit 3). And so on. There are 49 non-edge orbits for 3–5-node graphlets.

#### Node-pair-edge-GDV

Edge and non-edge orbits are mutually exclusive (Fig. 1). However, to perform LP, we need to contrast the topological neighborhood of nodes *v* and *u* against the neighborhood of nodes *s* and *t*, while hiding the information about whether *v* and *u* or *s* and *t* are actually linked. Hence, we need to reconcile edge orbits and non-edge orbits by defining *node-pair-GDV* to count the number of graphlets that a general *node pair*, which can be either an edge or a non-edge, touches at a given “node pair orbit”. For example, in Fig. 1, a node pair at node pair orbit 1 touches a triangle (graphlet 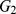) at edge orbit 2, if the node pair is an edge, or it touches a three-node path (graphlet 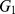) at non-edge orbit 1, if the node pair is a non-edge. Hence, we reconcile mutually exclusive edge orbit 2 and non-edge orbit 1 into a common node pair orbit 1. We do this for all edge- and non-edge orbits, resulting in 49 node pair orbits for 3–5-node graphlets.

#### Node-pair-GDV-centrality

We design *node-pair-GDV-centrality* to assign high centrality values to node pairs that participate in many graphlets. For nodes *v* and *u*, if 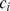 is the 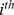 element of node-pair-GDV of the two nodes, then 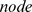-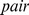-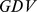-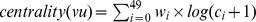. Thus, the more graphlets a node pair participates in, the higher its centrality. Note that we previously designed an analogous measure of the network centrality of a node, called node-GDV-centrality [50].

### Using the new measures for LP

Node-GDV-similarity and node-pair-GDV-centrality measures allow for several simple modifications which could perhaps improve LP accuracy, as follows.

#### Combining node-GDV-similarity and node-pair-GDV-centrality

Node-GDV-similarity favors linking topologically similar nodes. Node-pair-GDV-centrality favors linking nodes that share many graphlets. Combining the two would favor linking nodes that are *both* topologically similar and share many graphlets. We combine them as: 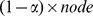-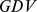-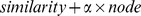-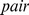-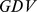-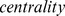. We vary *α* from 0 to 1 in increments of 0.2.

#### Prioritizing dense graphlets

Node-pair-GDV-centrality, as defined above, counts the number of graphlets that two nodes share, while assigning weights to different graphlets only with respect to “orbit dependencies” (see [43] for details). However, it ignores any information about the *denseness* of the graphlets that the two nodes share. Analogous to Adamic-Adar which favors some shared neighbors over others based on their degrees (see above), we might want to favor some shared graphlets over others based on their denseness. For example, it might be more reasonable to link two nodes that share many 4-node cliques than two nodes that share many 4-node paths. So, we favor denser shared graphlets over sparser shared graphlets by defining *density-weighted* (or simply *weighted*) *node-pair-GDV-centrality* (Section S1 in File S1). We evaluate both unweighted and weighted node-pair-GDV-centrality measures.

#### Graphlet size

To test how much of network topology is beneficial for LP, when using the graphlet-based measures, we use: 1) all 3–5-node graphlets, 2) 3–4-node graphlets, but not 5-node graphlets, and 3) only 3-node graphlets. Note that using the only 3-node graphlet within the node-pair-GDV-centrality at *α* of 1 (see above) is equivalent to the SN measure (see above). Hence, SN is a variation of node-pair-GDV-centrality. Also, note that when using 3-node graphlets, unweighted and weighted node-pair-GDV-centralities are equivalent. This is because there is only one 3-node graphlet when dealing with node-pair-GDVs, and its density is one. Determining which amount of topology to use is important: the more topology (the larger the graphlets), the higher the computational complexity. Exhaustive counting of all graphlets on up to *n* nodes in graph 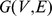 takes 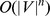; but, the practical running time is much smaller due to the sparseness of real networks [51]–[53]. Also, counting is embarrassingly parallel. Finally, fast non-exhaustive approaches exist for counting graphlets [53].

### Evaluation framework

We evaluate each of the existing and new LP methods on each of the PPI networks as follows.

First, we introduce synthetic noise in the given PPI network by randomly removing 5%–50% of its edges, with the goal of measuring how well the different methods can reconstruct the original network, using the original PPIs as the ground truth data. We apply the given LP measure to a “noisy” network created in this way and score each node pair in the network, so that the higher the score, the more likely the nodes are to be linked. We predict 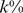 of the highest-scoring node pairs as edges. We vary *k* from 0% to 100% in increments of 1%. At each *k*, we count the number of true positives, true negatives, false positives, and false negatives, and we compute: 1) precision, recall, and F-score; and 2) sensitivity and specificity (Section S2 in File S1) [54]. For simplicity of comparing results across different methods, we summarize the performance of the methods over the entire range of *k* with respect to sensitivity and specificity by calculating the areas under receiver-operator curves (AUROCs), as well as with respect to precision and recall by calculating the areas under precision-recall curves (AUPRs).

To account for randomness in the above procedure, for each level of noise, we randomly remove the given percentage of edges from the original network five times and average the above statistics over the five runs. Ideally, we would perform more random runs, but this is impractical due to the required computational time. Plus, this might be unnecessary, since the standard deviations resulting from the five runs are typically very small (Section “Results and discussion”), and since even with five random runs of each method, we can compute the statistical significance of the difference in LP accuracy between a pair of methods by using the *paired t*-test. With this test, we compare five pairs of AUROCs corresponding to five random runs of two methods, and a low *p*-value would indicate that the null hypothesis (the difference between the accuracy of the two methods having a mean of 0) can be rejected.

Second, due to the availability of low-confidence PPI data for the HC network (see above), we perform an additional evaluation test: we apply the given LP method to the HC network and use the low-confidence PPIs as the ground truth data. We evaluate the method in the same way as above.

Third, we apply the given LP method to a network to de-noise it, and we evaluate the biological quality of the de-noised network with respect to the “enrichment” of predicted edges in Gene Ontology (GO) terms [38]. We compute the enrichment as the percentage of predicted edges, out of all edges in which both proteins have at least one GO term, in which the two end nodes share a GO term. As [36], we do this for biological process GO terms. To avoid potential biases, we consider only gene-GO term associations with experimental evidence codes. Since we de-noise networks by relying on their topology (i.e., on the PPIs), to avoid “circular arguments”, of these associations, we exclude associations inferred from PPIs. We compute the statistical significance of the enrichment by using the hypergeometric model (Section S2 in File S1).

Finally, we validate predicted edges absent from the original network by searching for them in an independent PPI data source. Here, we use BioGRID [5], because it is a trusted PPI data source. Again, we measure the statistical significance of validating the given number of predictions by using the hypergeometric model (Section S2 in File S1). We perform the external data source validation on AP/MS predictions as this network uses the same naming scheme as BioGRID.

## Results and Discussion

We study three yeast PPI networks: AP/MS, Y2H, and HC. We use a number of existing and new LP measures. The existing measures are degree product (DP), shared neighbors (SN), Jaccard coefficient (JC), Adamic-Adar (AA), Katz index (Katz), local path index (LPI), resource allocation index (RAI), and random walk with resistance (RWS). The new measures are node-GDV-similarity and node-pair-GDV-centrality. See Methods for details.

The two new graphlet-based measures allow us to address several important LP questions. First, we can combine node-GDV-similarity, which favors linking nodes with topologically similar neighborhoods, with node-pair-GDV-centrality, which favors linking nodes that share many graphlets and thus have large *extended* shared neighborhoods, to favor linking nodes that are *both* topologically similar and share many graphlets, which might be preferred. To test whether this is the case, we combine the two measures by varying the value of parameter *α* from 0 to 1, where *α* of 0 means that only node-GDV-similarity is used, and *α* of 1 means that only node-pair-GDV-centrality is used (see Methods). Second, we test whether favoring denser graphlets that are shared between the nodes in question within the node-pair-GDV-centrality measure is preferred over equally favoring all graphlets, independent on their density. We do this by evaluating both unweighted and weighted node-pair-GDV-centrality measures (see Methods). Third, to test how much of network topology is beneficial for LP, when using the graphlet-based measures, we use: 1) all 3–5 node graphlets, 2) only 3–4-node graphlets, and 3) only 3-node graphlets.

After we compare the different variations of graphlet-based measures, we evaluate the best of them against the existing LP measures. We perform three evaluation tests: 1) we introduce synthetic noise in the given PPI network by randomly removing a percentage of its edges, with the goal of measuring how well the given method can reconstruct the original network, using the original PPIs as the ground truth data; 2) given the availability of low-confidence PPI data corresponding to the HC network, we apply the given LP method to the original HC PPI network and use the corresponding low-confidence PPI data as the ground truth data when evaluating the method; and 3) we apply the LP methods to the original PPI networks to de-noise them, and we evaluate biological quality of the de-noised networks. In the first two evaluation tests above, we use systematic AUROC and AUPR frameworks as the evaluation criteria (see Methods). In the third evaluation test above, we compute the “enrichment” of predicted edges in GO terms [38], and also, we validate predicted edges in an external data source (see Methods).

Ultimately, we are less focused on identifying a superior LP method but more on testing whether we can de-noise a network so that the de-noised network is biologically more meaningful than the original one, as well as on which topological properties affect LP accuracy.

### Test 1: evaluating LP methods by introducing synthetic noise into PPI networks

Current PPI networks are noisy. The correct and complete ground truth interactomes are unknown. Thus, an alternative ground truth data has to be sought. We create synthetic ground truth data from the real PPI networks. For each PPI network, we add synthetic noise to the network by randomly removing 5%, 10%, 15%, 20%, 25%, and 50% of the original edges. Then, we evaluate the given LP method by applying it to a synthetically noised network and by measuring how well it reconstructs the original network (see Methods). Below, we initially discuss our results in the context of AUROCs, and later on we contrast these results against those returned by AUPRs.

#### Combining topological similarity and centrality of nodes to be linked improves LP accuracy

**Figure 2:**
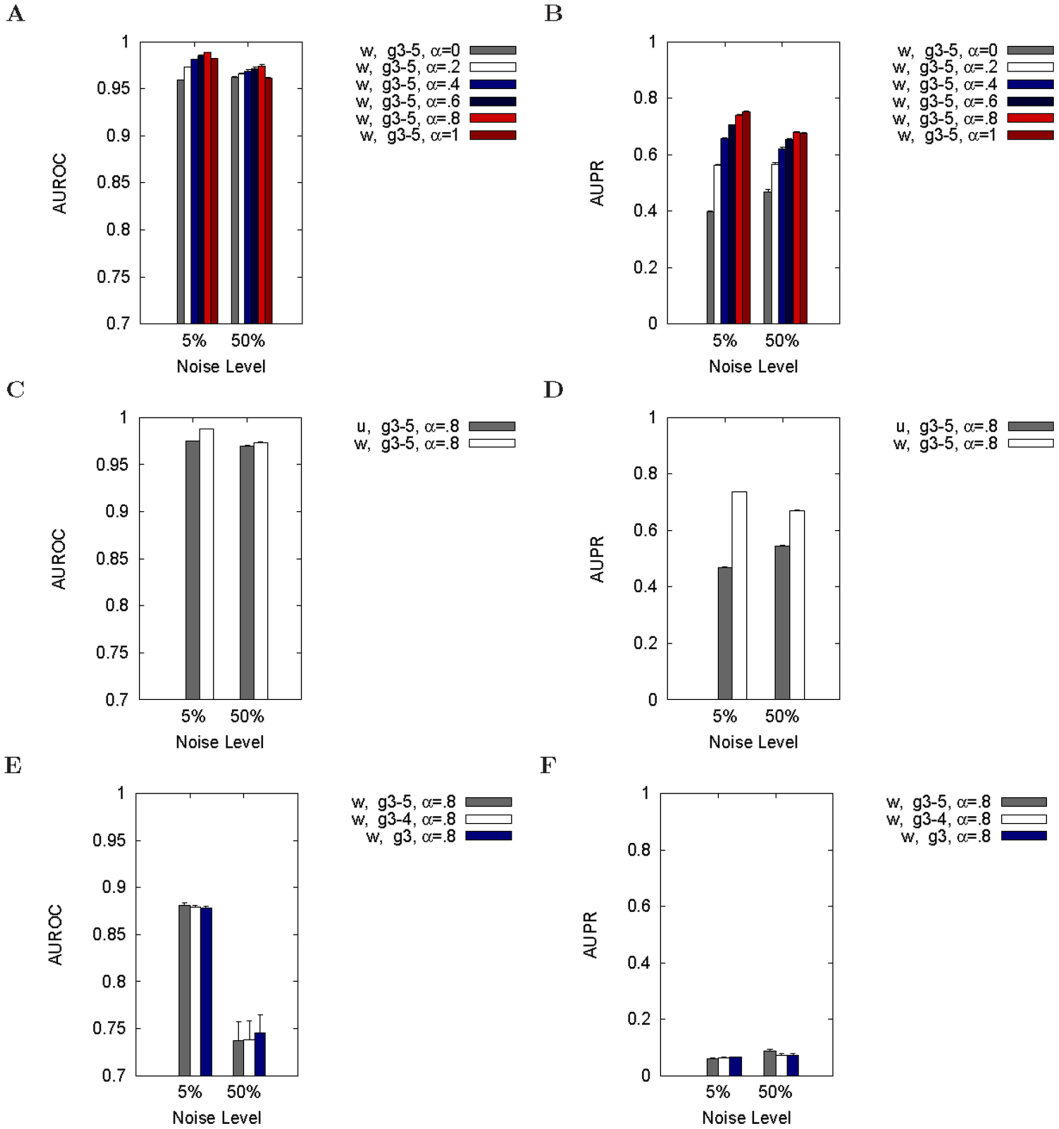
LP accuracy of the graphlet-based methods in the context of evaluation test 1. The accuracy is shown in terms of AUROCs (panels **A**, **C**, and **E**) as well as AUPRs (panels **B**, **D**, and **F**) at the lowest noise level of 5% and the highest noise level of 50% when comparing: 1) varying 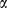
s in the AP/MS network (panels **A** and **B**); 2) unweighted (“u”) vs. weighted (“w”) node-pair-GDV-centrality in the HC network (panels **C** and **D**); and 3) different graphlet sizes (3–5-node (“g3–5”), 3–4-node (“g3–4”), and 3-node (“g3”) graphlets) in the Y2H network (panels **E** and **F**). Note that we intentionally vary the networks between the panels (AP/MS in panels **A** and **B**, HC in panels **C** and **D**, and Y2H in panels **E** and **F**), but only in order to represent each of the three studied networks equally; we show full results throughout File S1.

By combining node-GDV-similarity and node-pair-GDV-centrality with parameter *α* (see Methods), we find that nodes that are simultaneously topologically similar and share many graphlets are preferred for LP. In general, the larger the value of *α* (the more node-pair-GDV-centrality is used), the better the LP accuracy (Fig. 2 A). This suggests that the topological similarity of two nodes is less relevant for LP than the number of graphlets that the nodes share. However, using a small amount of node-GDV-similarity in the combined LP score (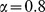) actually improves LP accuracy compared to using node-pair-GDV-centrality alone (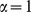), implying that topological similarity *is* relevant. The difference between LP accuracy of the best *α* of 0.8 and any other *α* in Fig. 2 A is statistically significant, with *p*-values below 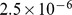 for 5% noise and below 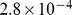 for 50% noise.

While the results in Fig. 2 A are for weighted node-pair-GDV-centrality, 3–5-node graphlets, two noise levels, and the AP/MS network, in general, they also hold for unweighted node-pair-GDV-centrality, all graphlet sizes, all noise levels, and HC and Y2H networks (Fig. S1 and S2 in File S1). And since 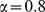 is statistically significantly superior to all other 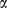
s, in the rest of the section, we focus only on this value of 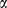.

#### Favoring denser shared graphlets improves LP accuracy

We find that preferring denser graphlets (see Methods) improves the LP performance: weighted node-pair-GDV-centrality outperforms the unweighted version (Fig. 2 C), and its superiority is statistically significant, with 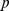-value of 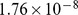 for 5% noise and 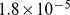 for 50% noise.

While the results in the figure are for all 3–5-node graphlets, two noise levels, and the HC network, in general, they also hold for all graphlet sizes, all noise levels, and AP/MS and Y2H networks (Fig. S3 in File S1). Thus, henceforth, we focus only on the superior weighted version of node-pair-GDV-centrality.

#### Using more topology does not always guarantee higher LP accuracy

There is no clear trend on how much topology is best (Fig. 2 E). For example, in the figure, for the lowest noise level of 5%, using 3–5-node graphlets is statistically significantly superior over using 3–4-node or 3-node graphlets, with 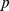-values of 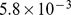 and 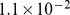, respectively. On the other hand, for the highest noise level of 50%, using 3-node graphlets is marginally superior over using 3–4-node graphlets, with 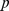-value of 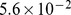, and it is statistically significantly superior over using 3–5-node graphlets, with 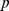-value of 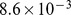. Hence, using more network topology can improve LP accuracy, but it is not guaranteed to do so.

Whereas the results in Fig. 2 E are only for two noise levels and the Y2H network, in general, they also hold for other noise levels and for AP/MS and HC networks (Fig. S4 in File S1).

Because in some cases using only 3-node graphlets is superior, and because the existing shared neighbors-based methods and SN in particular also rely on 3-node graphlets (see Methods), one might incorrectly assume that in these cases, our graphlet-based methods do not improve upon the existing SN method. However, it is at 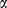 of 1 when our node-pair-GDV-centrality and existing SN method are equivalent (see Methods). Since our results at 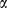 of 0.8 are superior over results at 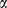 of 1 (see above), and since at 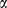 of 0.8 SN is actually combined with graphlet information encoded in the node-GDV-similarity measure, node-GDV-similarity actually *improves* the accuracy of SN even when using 3-node graphlets only and especially when using all 3–5-node graphlets is superior to using only 3-node graphlets.

Also, using deeper network topology is superior to using only the direct network neighborhood of nodes to be linked in the sense that node-GDV-similarity alone (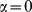) is superior to the existing DP method (Fig. S5 in File S1). This is interesting because the two methods are somewhat similar. They both take into account graphlet degrees of two nodes in question. They differ in that DP considers only the 2-node graphlet and hence captures only the direct (1-deep) network neighborhoods of the nodes, whereas node-GDV-similarity considers all 2–5-node graphlets, thus capturing up to 4-deep node neighborhoods. Hence, in this context, including more network topology helps. (We compare the existing methods to our new graphlet-based methods in more detail in the following section.)

In general, using larger graphlets can increase LP accuracy (the following sections also confirm this). Since counting larger graphlets is computationally expensive compared to counting smaller graphlets (see Methods), whether it is worth including the extra topological information that is captured by the larger graphlets depends on how significant the improvement is.

#### New graphlet-based measures are superior to the majority of the existing measures

Having examined different variations of the graphlet-based measures, we now compare these measures with the existing ones. Of all graphlet-based variations, we report the weighted version at 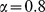 when considering 3–5-node graphlets, since this version generally performs the best. The existing measures include DP, SN, JC, AA, Katz, LPI, RAI, and RWS.

**Figure 3:**
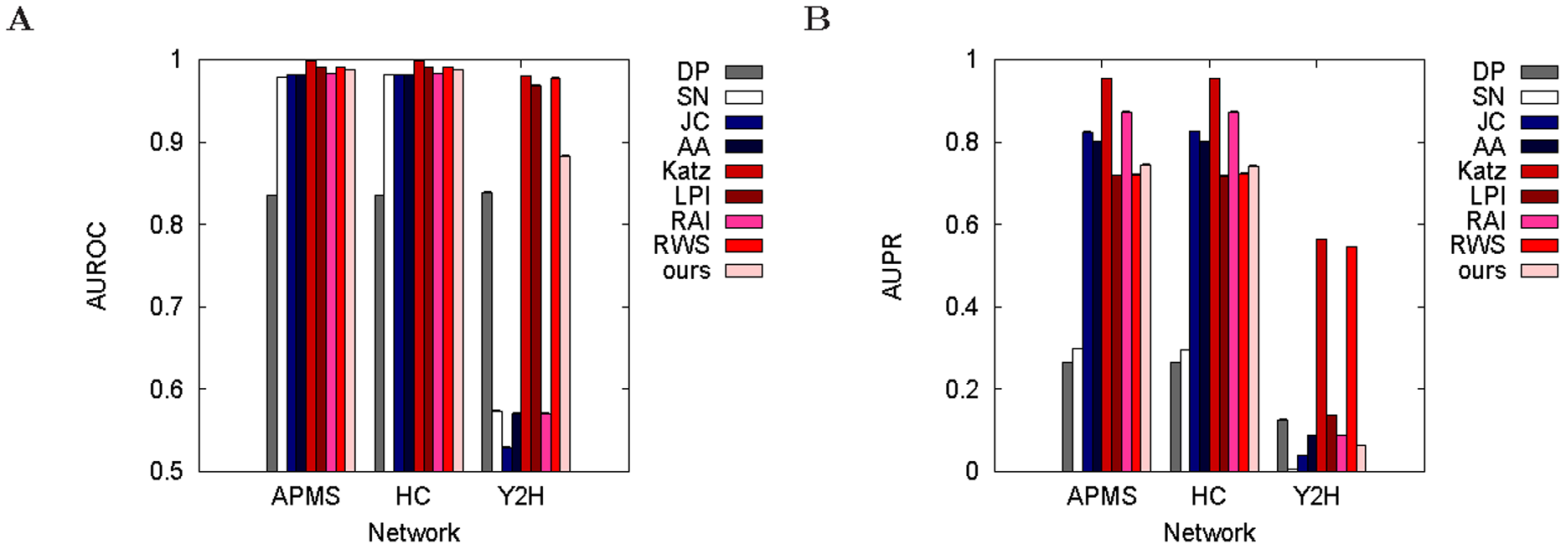
Comparison of different methods in the context of evaluation test 1. Our best method (“ours”) is compared against existing methods (DP, SN, JC, AA, Katz, LPI, RAI, and RWS) in terms of AUROCs (panel **A**) and AUPRs (panel **B**) for synthetically noised AP/MS, HC, and Y2H networks at 5% noise level. Here, “ours” corresponds to using 3–5-node weighted graphlets at 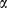 = 0.8. Results for all other noise levels are shown throughout File S1.

Our graphlet-based method is superior to five out of the eight existing methods in all three networks (Fig. 3 A). These five methods are: DP, SN, JC, AA, and RAI. And whereas Katz, LPI, and RWS are somewhat superior to our graphlet-based method in this evaluation test and with respect to the evaluation criterion from Fig. 3 A (namely AUROCs), we show later that our method beats each of Katz, LPI, and RWS in at least one different evaluation test or with respect to at least one other evaluation criterion (such as AUPRs or biological correctness of de-noised networks; see below). The 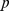-value of the difference between LP accuracy of our method and any of the five inferior methods in Fig. 3 A is below 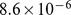, 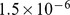, and 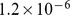 for AP/MS, HC, and Y2H networks, respectively. It is worth noting that most of the methods perform quite well, reaching very high AUROCs of up to 0.998, 0.998, and 0.98 in AP/MS, HC, and Y2H networks, respectively. Whereas Fig. 3 A is for the lowest noise level, the results are similar for other noise levels (Fig. S5 in File S1). Interestingly, our graphlet-based measures further improve over the existing measures as the noise increases.

#### ROCs vs. precision-recall curves

Thus far, we have shown AUROC results. AUROCs are commonly used to evaluate methods over the entire [0%,100%] range of *k* [21] (see Methods, as well as Fig. S1–S8 in File S1). In a similar fashion, AUPRs can be computed (see Methods, as well as Fig. S9–19 in File S1). Whereas one would hope that different evaluation criteria such as AUROCs and AUPRs would identify the same methods as best performing, we actually find that the AUROC results are not necessarily consistent with AUPR results.

Namely, while in both AP/MS and HC networks our best graphlet-based measure is better than JC, AA, and RAI with respect to AUROCs (along with some other existing measures) (Fig. 3 A; Fig. S5 in File S1), JC, AA, and RAI are better with respect to AUPRs (Fig. 3 B; Fig. S13 in File S1). On the other hand, whereas Katz, LPI, and RWS are better than our graphlet-based method with respect to AUROCs (Fig. 3 A), our graphlet-based method is better than LPI and RWS with respect to AUPRs (Fig. 3 B). Note that while Katz remains superior to our graphlet-based methods with respect to AUPRs, as we show later, Katz becomes inferior to our graphlet-based methods with respect to an alternative evaluation criterion, namely biological correctness of de-noised networks (see below).

Further, for Y2H, we notice a different inconsistency: whereas AUROCs are high for the best-performing methods (Fig. S5 in File S1), AUPRs indicate poor performance of almost all methods, as precision is always relatively low (Fig. S13 in File S1).

Even though optimizing AUROCs does not necessarily optimize AUPRs [54], the observed inconsistencies are alarming, and the LP community needs to be aware. We address this issue by also comparing the different methods with respect to biological correctness of their de-noised networks (see below). But first, we check whether results depend on the ground truth data, as follows.

### Test 2: evaluating LP methods on HC network with respect to low-confidence PPI data

**Figure 4:**
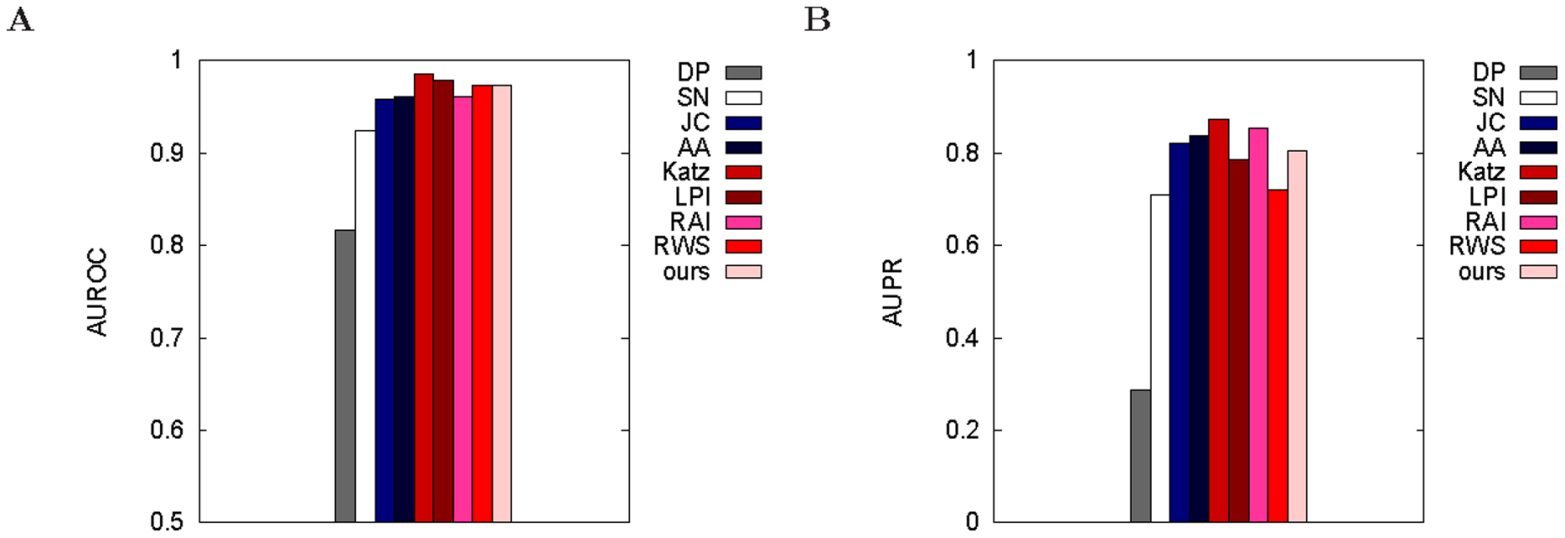
Comparison of different methods in the context of evaluation test 2. Our best method (“ours”) is compared against existing methods (DP, SN, JC, AA, Katz, LPI, RAI, and RWS) in terms of AUROCs (panel **A**) and AUPRs (panel **B**) for the HC network when using low confidence PPIs as the ground truth data. Here, “ours” corresponds to using 3–4-node weighted graphlets at 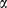 = 0.4.

When we evaluate the LP methods on the HC network with low-confidence PPIs as the ground truth data, we find that:

1. As in the previous section (when evaluating LP methods by introducing synthetic noise into PPI networks), combining topological similarity and centrality of nodes to be linked improves LP accuracy. However, now 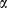 of 0.4 is the best overall instead of 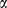 of 0.8 (Fig. S20 and S21 in File S1): topological similarity is now *more* relevant than the number of shared graphlets.
2. As in the previous section, favoring denser graphlets improves LP accuracy for the best 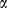 (Fig. S22 in File S1).
3. As in the previous section, using more topology can improve LP accuracy (Fig. S23 in File S1). Using 3–4-node graphlets at the best 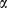 of 0.4 results in higher AUROC than using only 3-node graphlets at *any* 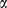, and using 3–5-node graphlets at 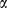 of 0.8 results in higher AUROCs than using only 3-node graphlets or 3–4-node graphlets at the same 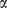 (Fig. S23 in File S1).
4. As in the previous section, our best graphlet-based measure in this context (using 3–4-node weighted graphlets at 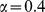) is superior to the majority (namely six) of the eight existing measures (Fig. 4 A; Fig. S24 in File S1). The only superior methods in this context (namely with respect to AUROCs) are Katz and LPI, but as we show later, these two methods are inferior to our graphlet-based method with respect to at least one alternative evaluation criterion.
5. We again see inconsistencies between AUROC and AUPR results (Fig. 4 A and B; Fig. S25–S29 in File S1). For example, whereas LPI is superior to our graphlet-based method with respect to AUROCs, LPI is inferior to our graphlet-based method with respect to AUPRs.

### Test 3: de-noising the PPI networks

Since both our new and the existing methods perform well on all networks with respect to AUROCs (Fig. 3 A and Fig. 4 A; Fig. S5 in File S1), we use the overall best graphlet-based method (weighted 3–5-node graphlets at 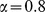) as well as DP, SN, JC, AA, Katz, LPI, RAI, and RWS and to de-noise the networks. We score each node pair in a network, and we predict as edges in the de-noised network the top 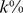 highest scoring node pairs. We choose 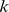 so that the number of edges in the de-noised network matches the number of edges in the original network. Depending on the network, 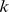 falls between 1% and 2%. We choose 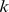 in this way because most of the methods achieve the maximum F-score in this range of 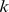 (Fig. S17–S19 in File S1).

#### Gene Ontology (GO) validation of de-noised networks

**Figure 5:**
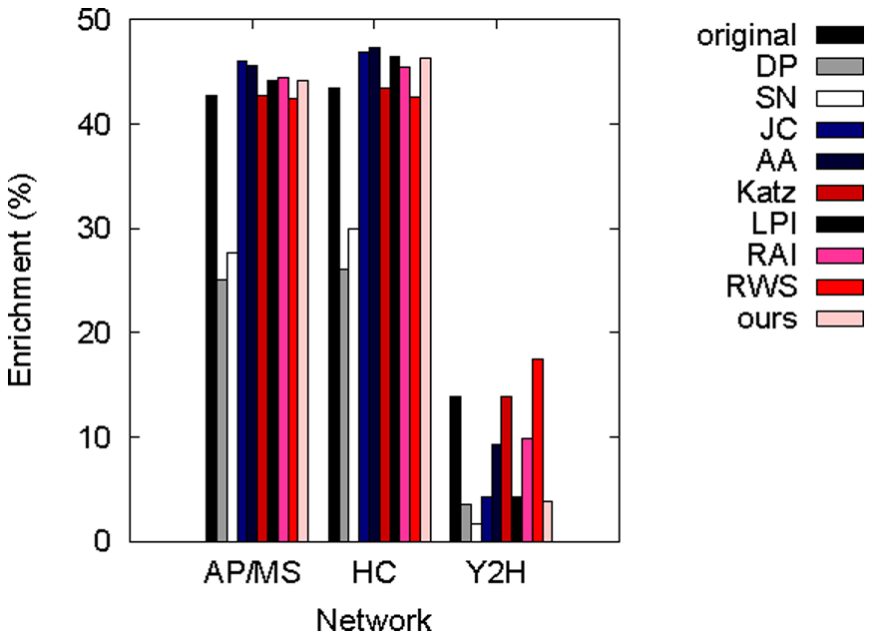
Comparison of different methods in the context of evaluation test 3. Our best method (“ours”) is compared against existing methods (DP, SN, JC, AA, Katz, LPI, RAI, and RWS) in terms of GO enrichments of AP/MS, HC, and Y2H networks and their de-noised counterparts. Here, “ours” corresponds to using 3–5-node weighted graphlets at *α* = 0.8.

We validate biological correctness of the de-noised networks by computing the enrichment of all predicted edges in GO terms (see Methods and Fig. 5). When we de-noise AP/MS and HC networks, the enrichment is statistically significant for all methods (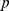-values 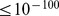). Our method as well as JC, AA, LPI, and RAI *improve* the quality of the original AP/MS and HC networks. This is important, since the main goal of LP is to de-noise a network so that the de-noised network is more meaningful than the original one. While the GO enrichments are worse for the de-noised networks than for the original Y2H network for all methods but RWS, the enrichments are still statistically significant (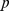-values≤0.05) for our method, as well as for all existing methods except SN.

Importantly, in this evaluation test and with respect to this evaluation criterion, Katz, which is the only method that is superior to our best graphlet-based method with respect to both AUROCs and AUPRs, now loses its superiority: our best graphlet-based method now beats Katz in AP/MS and HC networks. This not only verifies that our method is superior to every one of the eight existing methods with respect to at least one evaluation criterion (be it AUROCs, AUPRs, or biological correctness of de-noised networks), but it also implies an additional inconsistency between the different evaluation criteria regarding the best-performing method(s). As a further illustration of this inconsistency, we note that JC and AA, which are not superior to all other measures with respect to either AUROCs or AUPRs, now slightly outperform all of the other measures for AP/MS and HC networks (Fig. 5).

#### Intersection of de-noised networks produced by different methods

Since we de-noise a network with multiple LP methods, we measure the intersections between the de-noised networks (Fig. S30 in File S1). The intersections are quite large between our method on one side and the shared neighbors-based methods or LPI on the other. JC is an exception, as it is somewhat different not only from our method but also from other shared neighbors-based methods. Actually, our method is more similar to SN and AA than JC is. The similarity between LPI and our method, as well as between LPI and the shared-neighbors-based methods (excluding JC) is not surprising, as all of these measures are intuitively similar (see Methods). In terms of the intersections between the original networks on one hand and de-noised networks for the different methods on the other, the intersections are the largest for Katz, followed by a number of more-less tied methods (including ours), followed by DP (Fig. S30 in File S1).

It is important to note that de-noised AP/MS and HC networks resulting from Katz (which achieves extremely high AUROCs and AUPRs in AP/MS and HC networks in evaluation tests 1 and 2) are completely identical to the original AP/MS and HC networks. That is, when used to de-noise these networks, Katz cannot generate any new edges or remove any of the existing ones (and hence the maximum overlap with the original networks in Fig. S30 in File S1). And while on one hand one might argue that because of high AUROCs and AUPRs Katz is very accurate, on the other hand, Katz is clearly incapable of de-noising real-world networks, which could be viewed as its disadvantage.

#### Validation of de-noised networks on external PPI data

We aim to validate “new predicted edges” (predicted edges not present in the original network; Table S1 in File S1) by searching for them in BioGRID as an independent data source. We do this for the AP/MS network. Even though validation accuracy varies across the methods, all methods achieve statistically significant validation rates (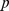-values below 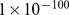), except Katz, which predicts no new edges and thus cannot be validated. Of the remaining existing methods, only RAI, JC, and RWS outperform our method (Fig. S31 in File S1).

## Concluding Remarks

We tackle the problem of link prediction (LP) in the context of PPI network de-noising. We comprehensively study what is it in the PPI network topology around nodes in question that dictates whether the nodes should be linked. To evaluate whether nodes that share many neighbors and are thus close in the network are favored over distant nodes (as is the assumption of most of the existing LP methods), whether topological similarity between nodes in question has any effect, and how much of the network topology should be included, we propose new LP methods, since none of the existing methods allowed for answering all of these questions. Unlike the existing methods, our new methods allow for combining topological similarity of the nodes to be linked with the information about the size of their shared neighborhood, while at the same time allowing to vary the amount of network topology that is taken into account for LP. We demonstrate via a thorough evaluation that our new methods outperform each of the eight existing methods with respect to at least one evaluation criterion. Importantly, when we use the LP methods to de-noise real-world PPI networks, we find that the de-noised networks improve biological correctness of the original networks, which is the ultimate goal of LP in computational biology.

## Supporting Information

File S1 **This file combines Sections S1–S2, Table S1, and Fig. S1–S31.**
(PDF)
