# Supplementary material for: Revealing Missing Parts of the Interactome via Link Prediction
Source: PLoS One. 2014 Mar 3;9(3):e90073. doi: 10.1371/journal.pone.0090073 (PMC3940777; doi:10.1371/journal.pone.0090073)
Supplement: File S1 — This file combines Sections S1–S2, Table S1, and Fig. S1–S31. (PDF) [file pone.0090073.s001.pdf]

Supporting Information S1: Revealing missing parts of the interactome via link prediction

Yuriy Hulovatyy, Ryan W. Solava, and Tijana Milenković*

Department of Computer Science and Engineering, ECK Institute for Global Health, and
Interdisciplinary Center for Network Science and Applications
University of Notre Dame, Notre Dame, IN 46556, USA

S1 MORE DETAILS ON DENSITY-WEIGHTED NODE-PAIR-GDV-CENTRALITY

As discussed in Section 2.3.3 of the main paper, we aim to favor denser shared graphlets over sparser shared graphlets, where we define the density of a graphlet as the percentage of edges present in the graphlet out of all possible edges, ignoring the node pair in question. Thus, we design *density-weighted node-pair-GDV-centrality*, as follows. If c_i is the ith element of node-pair-GDV of the nodes v and u, then we define density-weighted node-pair-GDV-centrality of v and u as: $\sum_{i=0}^{49} w_i \times (\frac{3}{2}d_i - \frac{1}{2})^{10} \times log(c_i + 1)$, where d_i is the density of the graphlet corresponding to node pair orbit i (and w_i is the weight that takes into account orbit dependencies, just as in the original definition). This is just one possible heuristic for favoring denser graphlets over sparser ones. In the formula, the density is multiplied by $\frac{3}{2}$ and then subtracted by $\frac{1}{2}$ to scale it to [0,1] interval. The scaled density is then raised to the power of 10 to favor higher density graphlets (especially cliques) over lower density graphlets exponentially rather than linearly.

S2 MORE DETAILS ON EVALUATION FRAMEWORK

As discussed in Section 2.4 of the main paper, we evaluate each of the existing and new LP methods on each of the PPI networks by computing the number of true positives (TP), true negatives (TN), false positives (FP), and false negatives (FN) predicted by the method. TP are predicted edges that are present in the original PPI network, TN are missed edges (i.e., predicted non-edges) that that are absent from the original PPI network, FP are predicted edges that are absent from the original PPI network, and FN are missed edges that are present in the original PPI network. Clearly, an ideal method would maximize TP and TN and minimize FP and FN over the entire range of k. We summarize the four statistics in two ways, by computing: 1) precision and recall, together with the corresponding F-score and 2) sensitivity and specificity, together with the corresponding receiver-operator curves (ROCs). Precision $= \frac{TP}{TP+FP}$. Recall $= \frac{TP}{TP+FN}$. As precision increases, recall decreases, and vice versa. To balance between the two measures, we combine them into popular F-score measure as: *F-score* $= 2 \cdot \frac{\text{Precision}\cdot\text{Recall}}{\text{Precision}+\text{Recall}}$. We plot precision-recall and F-score curves over the entire range of k. Sensitivity $= \frac{TP}{TP+FN}$. Specificity $= \frac{TN}{FP+TN}$. For simplicity of comparing results across different methods, we summarize the performance of the methods over the entire range of k with respect to sensitivity and specificity by calculating the areas under the ROCs (AUROCs).

After we evaluate the LP methods, we apply them to a PPI network to de-noise it, and we evaluate the biological quality of the de-noised network with respect to the “enrichment” of predicted edges in Gene Ontology (GO) terms. That is, we compute the enrichment as the percentage of predicted edges, out of all edges in which both proteins have at least one GO term, in which the two end nodes share a GO term. We compute the statistical significance of the given enrichment by calculating the probability (i.e., p-value) of observing the same or higher enrichment by chance using the hypergeometric model. Let us denote by N the set of pairs of proteins in the original PPI network where both proteins have at least one biological process GO term, by S the subset of protein pairs in N that have a GO term in common, by e the subset of protein pairs in N that are predicted as edges, and by k the subset of protein pairs in e that have a GO term in common. Then, the enrichment is $|k|/|e|$ and its p-value is: p-value $= 1 - \sum_{i=0}^{|k|-1} \frac{\binom{|S|}{i}\binom{|N|-|S|}{|e|-i}}{\binom{|N|}{|e|}}$. We use p-value threshold of 0.05.

Also, we validate predicted edges absent from the original network by searching for them in an independent PPI data source. We measure the statistical significance of validating the given number of predictions by using the above formula, except that now N is the set of all pairs of proteins in the PPI network in which both proteins are present in the BioGRID data, S is the subset of protein pairs in N that interact in BioGRID, e is the subset of protein pairs in N that are new predicted edges, and k is the subset of protein pairs in e that interact in BioGRID. We use p-value threshold of 0.05.

*To whom correspondence should be addressed

Table S1. The list of all node pairs predicted as edges by any method. Columns 1 and 2 correspond to the node names. Column 3 corresponds to the number of methods that predict the given node pair as an edge. Column 4 contains a "1" if the node pair is a "new predicted edge" (predicted edge not present in the original network) and a "0" otherwise. Columns 5-9 list the rankings of the given node pair by the different methods (the same methods as in Fig. 3(A) of the main paper); if the given node pair is not predicted as an edge by a given method, a '-' appears. The three tabs in the file correspond to the three analyzed networks: AP/MS, HC, and Y2H.

http://www3.nd.edu/~cone/LP/ST1.xlsx

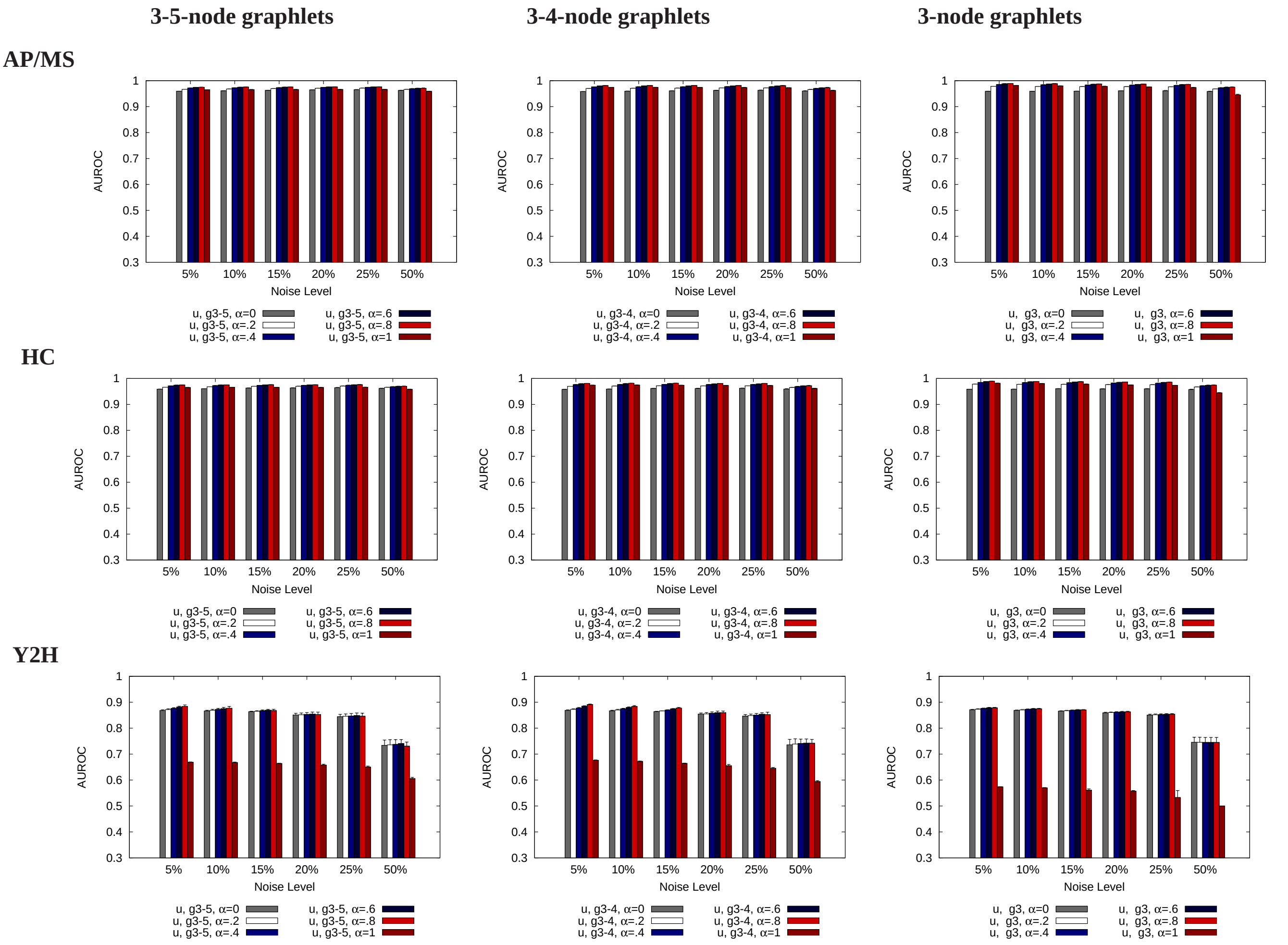

Fig. S1. Link prediction accuracy of the **unweighted** graphlet-based methods in terms of **AUROCs** at different noise levels as α is varied from 0 to 1. Recall that $\alpha = 0$ corresponds to using node-GDV-similarity alone and $\alpha = 1$ corresponds to using node-pair-GDV-centrality alone. The first column corresponds to using 3-5-node graphlets, the second column corresponds to using 3-4-node graphlets, and the third column corresponds to using 3-node graphlets only. The first row corresponds to the AP/MS network, the second row corresponds to the HC network, and the third row corresponds to the Y2H network. The error bar for a given method corresponds to the standard deviation over five random runs of the method. In some cases, standard deviations are so small that they are not visible.

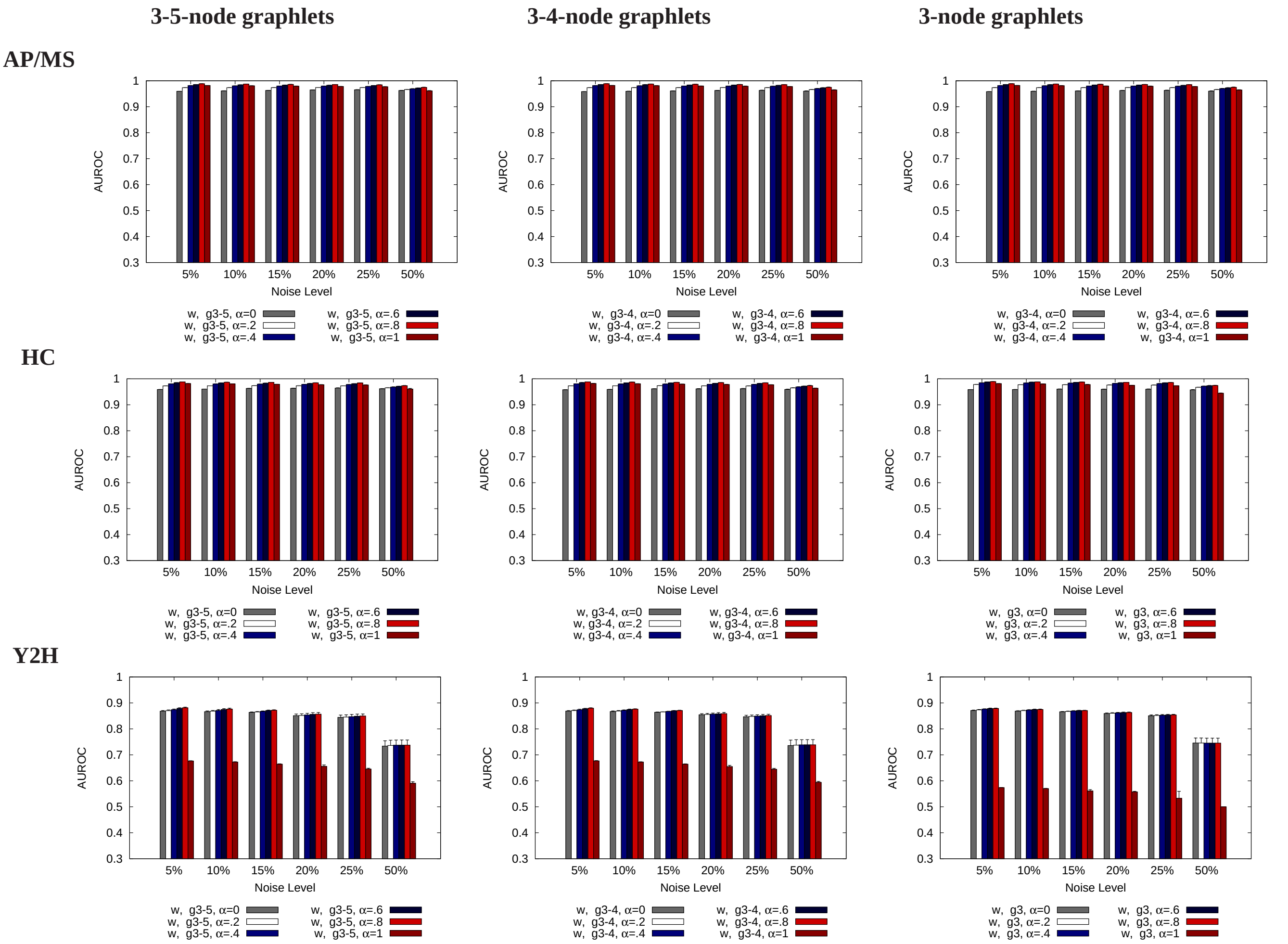

Fig. S2. Link prediction accuracy of the **density-weighted** graphlet-based methods in terms of **AUROCs** at different noise levels as α is varied from 0 to 1. Recall that $\alpha = 0$ corresponds to using node-GDV-similarity alone and $\alpha = 1$ corresponds to using node-pair-GDV-centrality alone. The first column corresponds to using 3-5-node graphlets, the second column corresponds to using 3-4-node graphlets, and the third column corresponds to using 3-node graphlets only. The first row corresponds to the AP/MS network, the second row corresponds to the HC network, and the third row corresponds to the Y2H network. The error bar for a given method corresponds to the standard deviation over five random runs of the method. In some cases, standard deviations are so small that they are not visible.

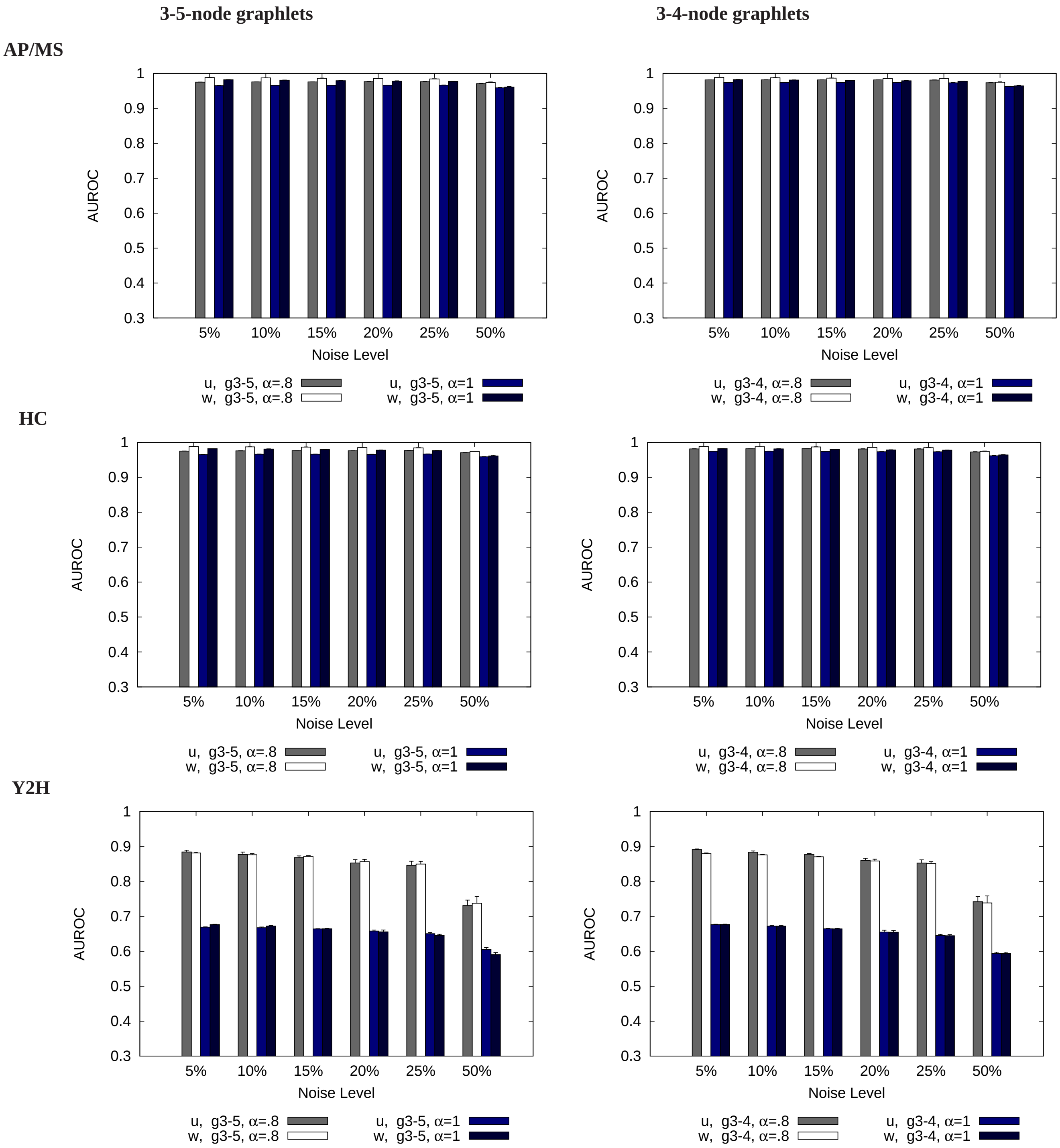

Fig. S3. Link prediction accuracy of the graphlet-based methods in terms of **AUROCs** at different noise levels **comparing unweighted and weighted versions of the methods**. The first column corresponds to using 3-5-node graphlets and the second column corresponds to using 3-4-node graphlets. The results for using 3-node-graphlets are not included, as for this graphlet size, the unweighted and weighted versions of the methods are equivalent. Also, unlike in the following figures, the results for α of 0 are not included in this figure, as at this α, the unweighted and weighted versions of the methods are equivalent. The first row corresponds to the AP/MS network, the second row corresponds to the HC network, and the third row corresponds to the Y2H network. The error bar for a given method corresponds to the standard deviation over five random runs of the method. In some cases, standard deviations are so small that they are not visible.

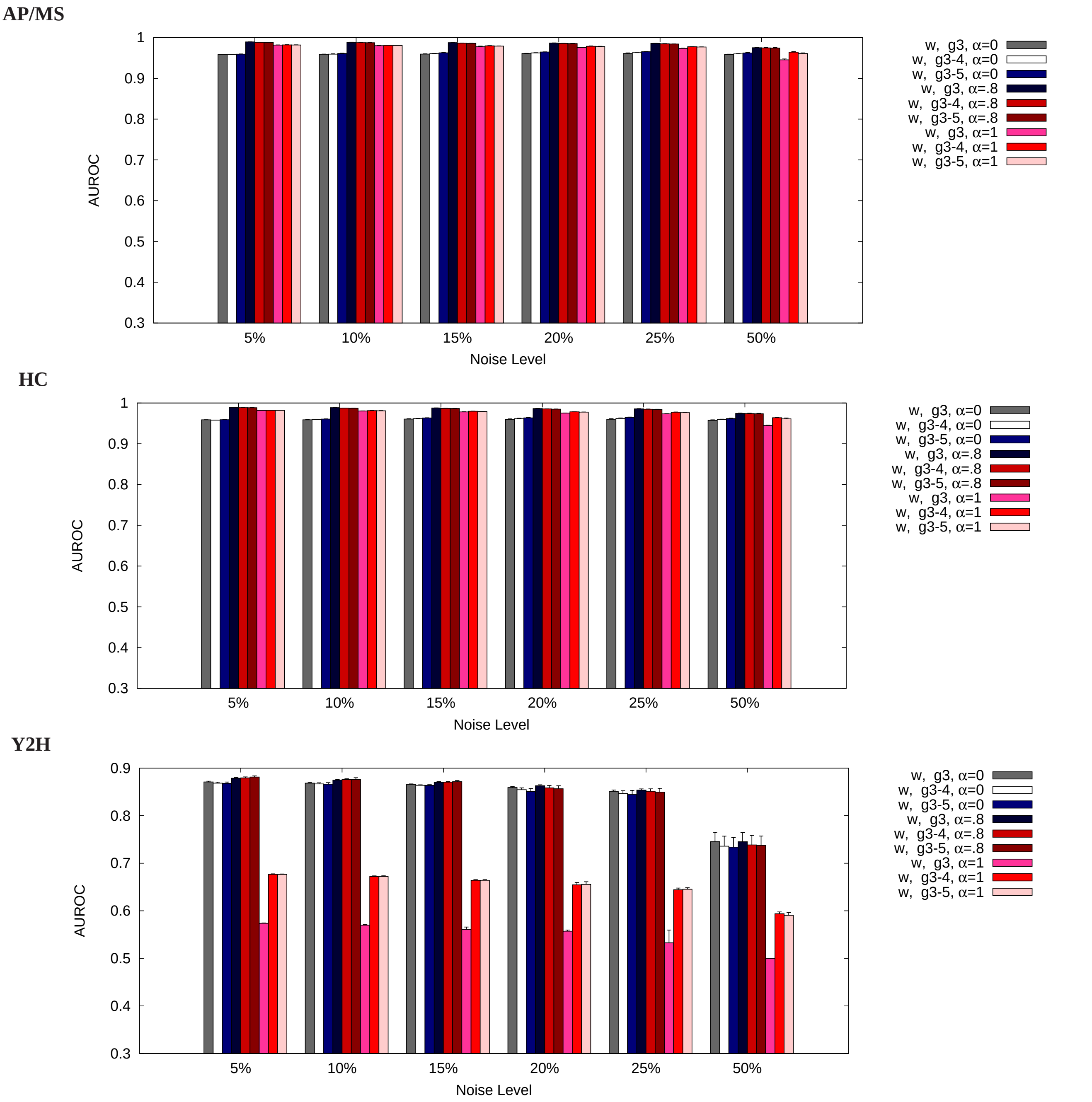

Fig. S4. Link prediction accuracy of the weighted graphlet-based methods in terms of **AUROCs** at different noise levels **comparing different graphlet sizes** in each of the three networks (AP/MS, HC, and Y2H). The error bar for a given method corresponds to the standard deviation over five random runs of the method. In some cases, standard deviations are so small that they are not visible.

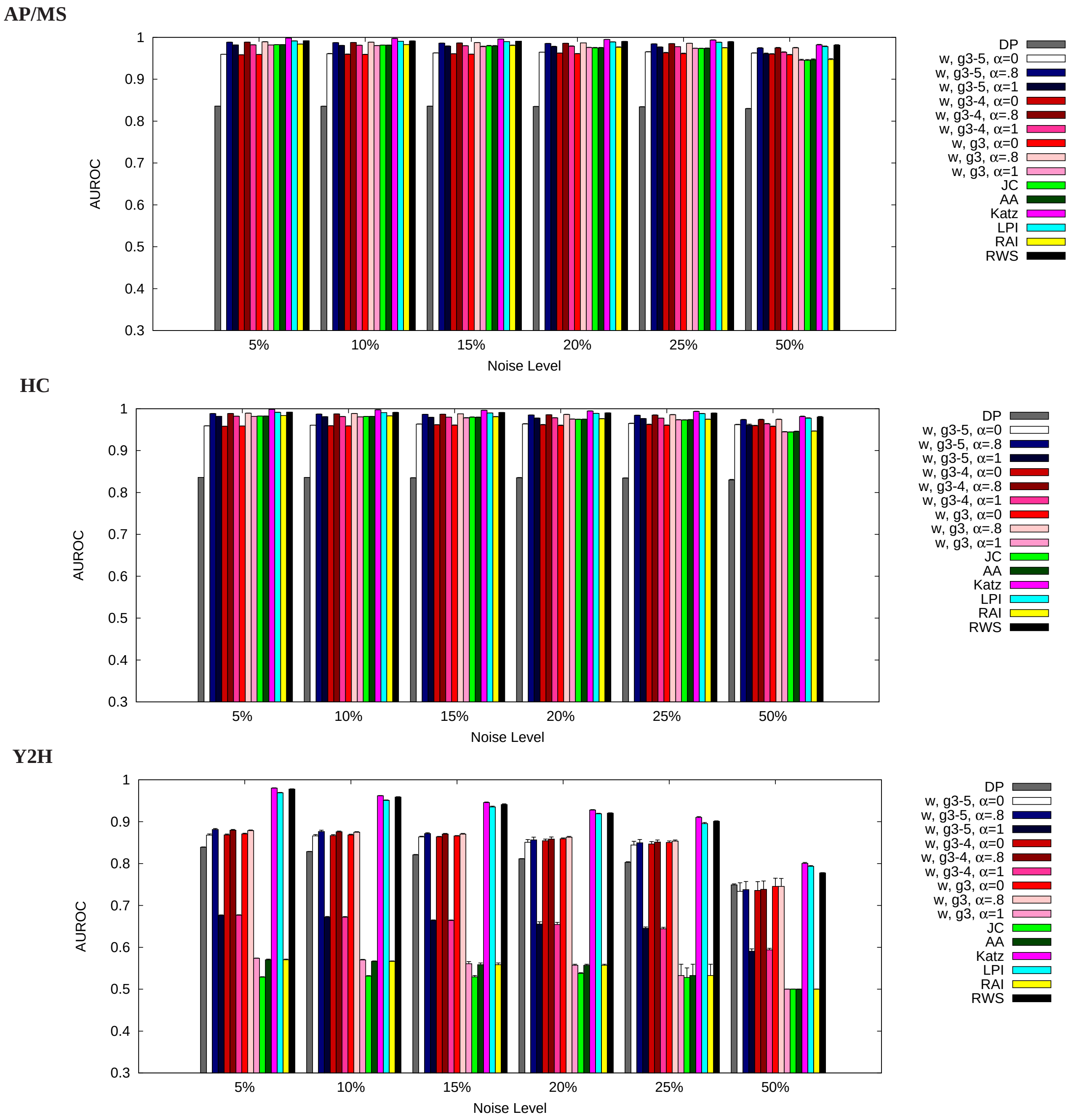

Fig. S5. Link prediction accuracy of our new methods and existing methods in terms of **AUROCs** at different noise levels **comparing the different methods** in each of the three networks (AP/MS, HC, and Y2H). The error bar for a given method corresponds to the standard deviation over five random runs of the method. In some cases, standard deviations are so small that they are not visible.

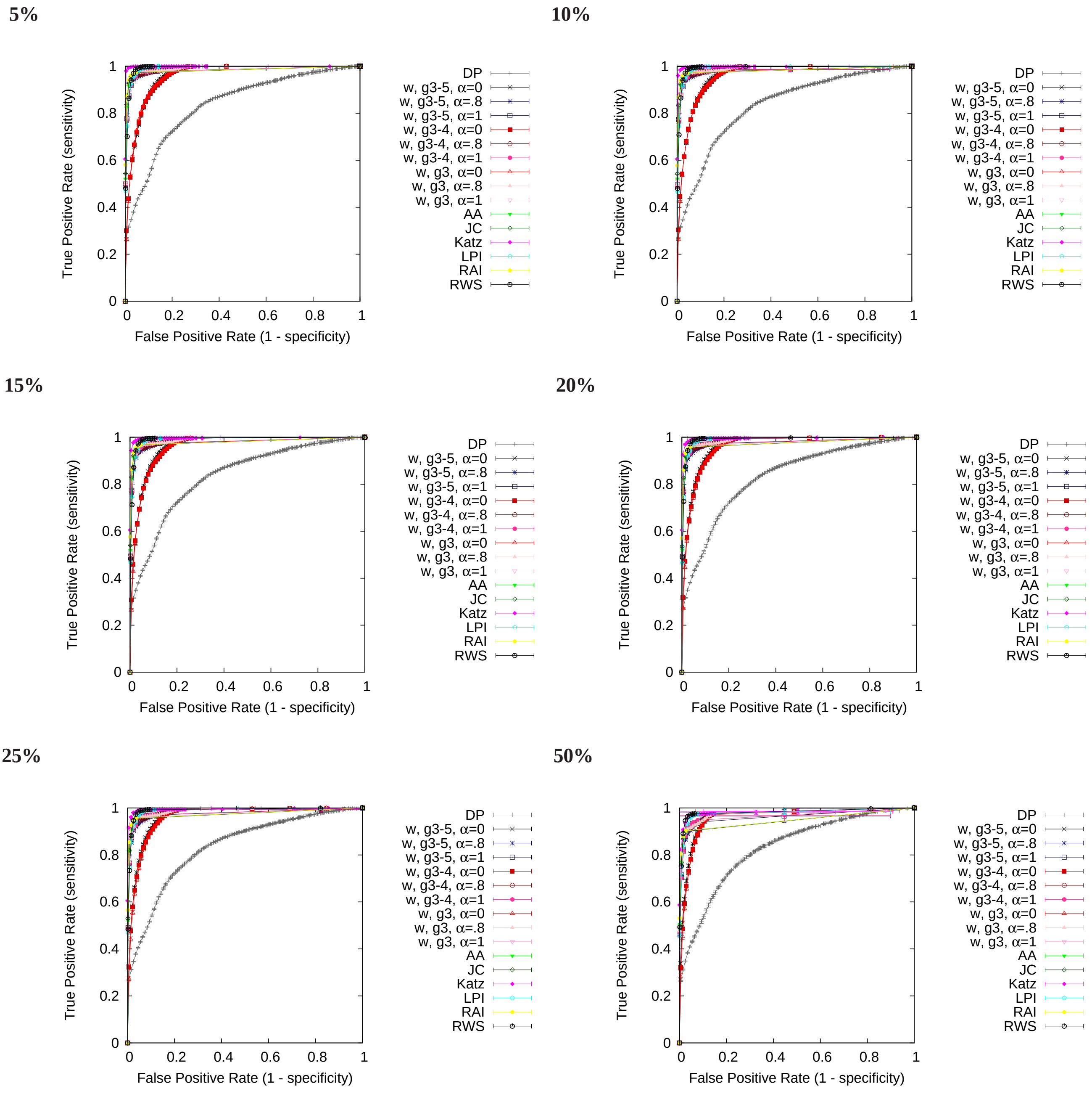

Fig. S6. Receiver operator curves for the AP/MS network for each noise level. The vertical error bar at a given value of k and for a given method corresponds to the standard deviation of true positive rate over five random runs of the method. The horizontal error bar at a given value of k and for a given method corresponds to the standard deviation of false negative rate over five random runs of the method.

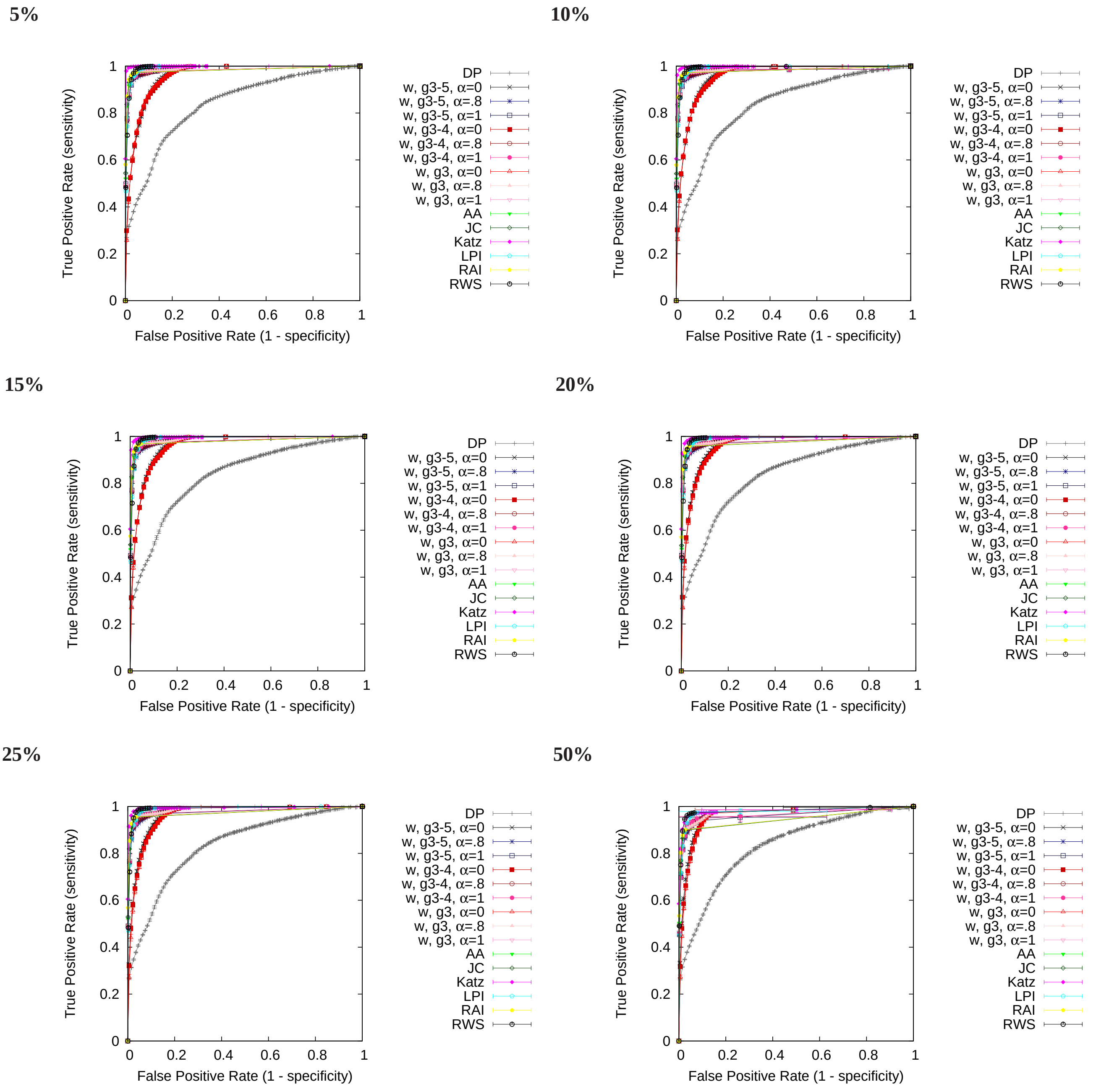

Fig. S7. Receiver operator curves for the HC network for each noise level. The vertical error bar at a given value of k and for a given method corresponds to the standard deviation of true positive rate over five random runs of the method. The horizontal error bar at a given value of k and for a given method corresponds to the standard deviation of false negative rate over five random runs of the method.

5%

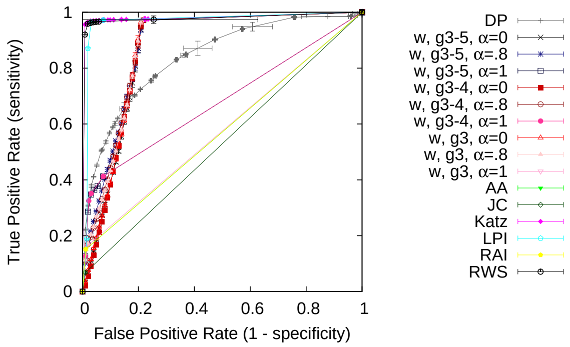

10%

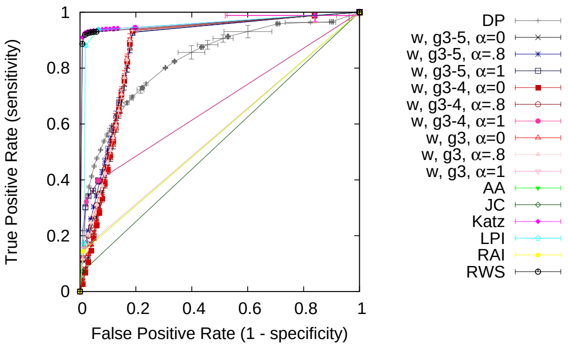

15%

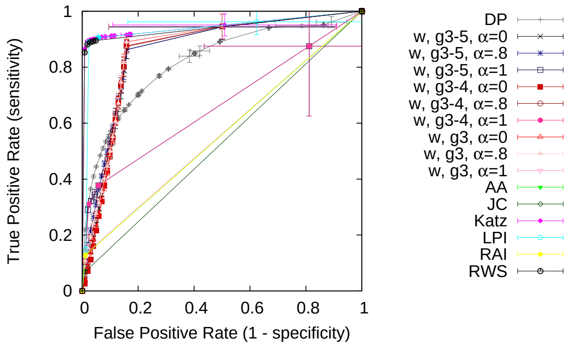

20%

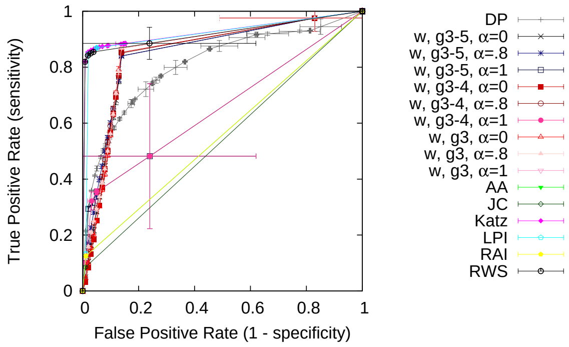

25%

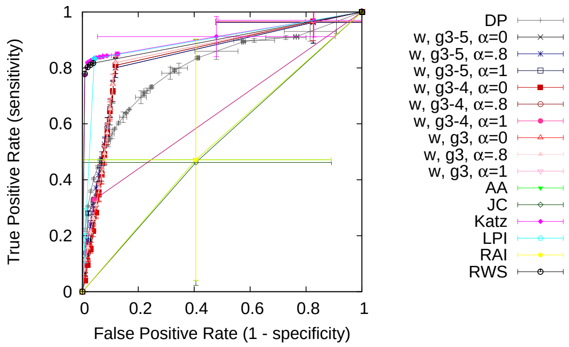

50%

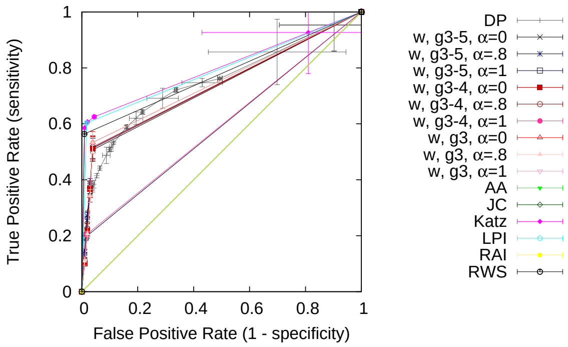

Fig. S8. Receiver operator curves for the Y2H network for each noise level. The vertical error bar at a given value of k and for a given method corresponds to the standard deviation of true positive rate over five random runs of the method. The horizontal error bar at a given value of k and for a given method corresponds to the standard deviation of false negative rate over five random runs of the method.

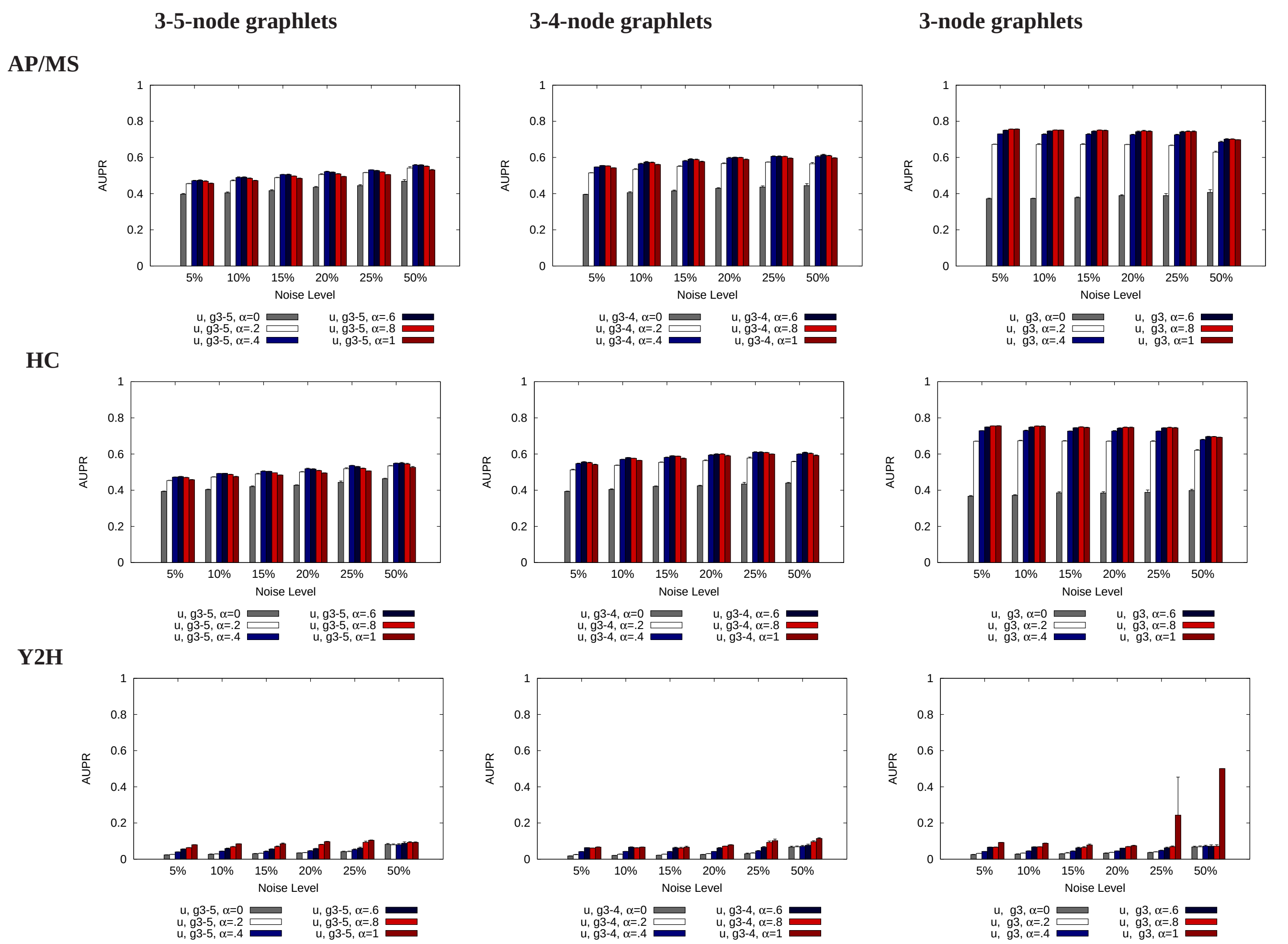

Fig. S9. Link prediction accuracy of the **unweighted** graphlet-based methods in terms of **AUPRs** at different noise levels as α is varied from 0 to 1. Recall that $\alpha = 0$ corresponds to using node-GDV-similarity alone and $\alpha = 1$ corresponds to using node-pair-GDV-centrality alone. The first column corresponds to using 3-5-node graphlets, the second column corresponds to using 3-4-node graphlets, and the third column corresponds to using 3-node graphlets only. The first row corresponds to the AP/MS network, the second row corresponds to the HC network, and the third row corresponds to the Y2H network. The error bar for a given method corresponds to the standard deviation over five random runs of the method. In some cases, standard deviations are so small that they are not visible.

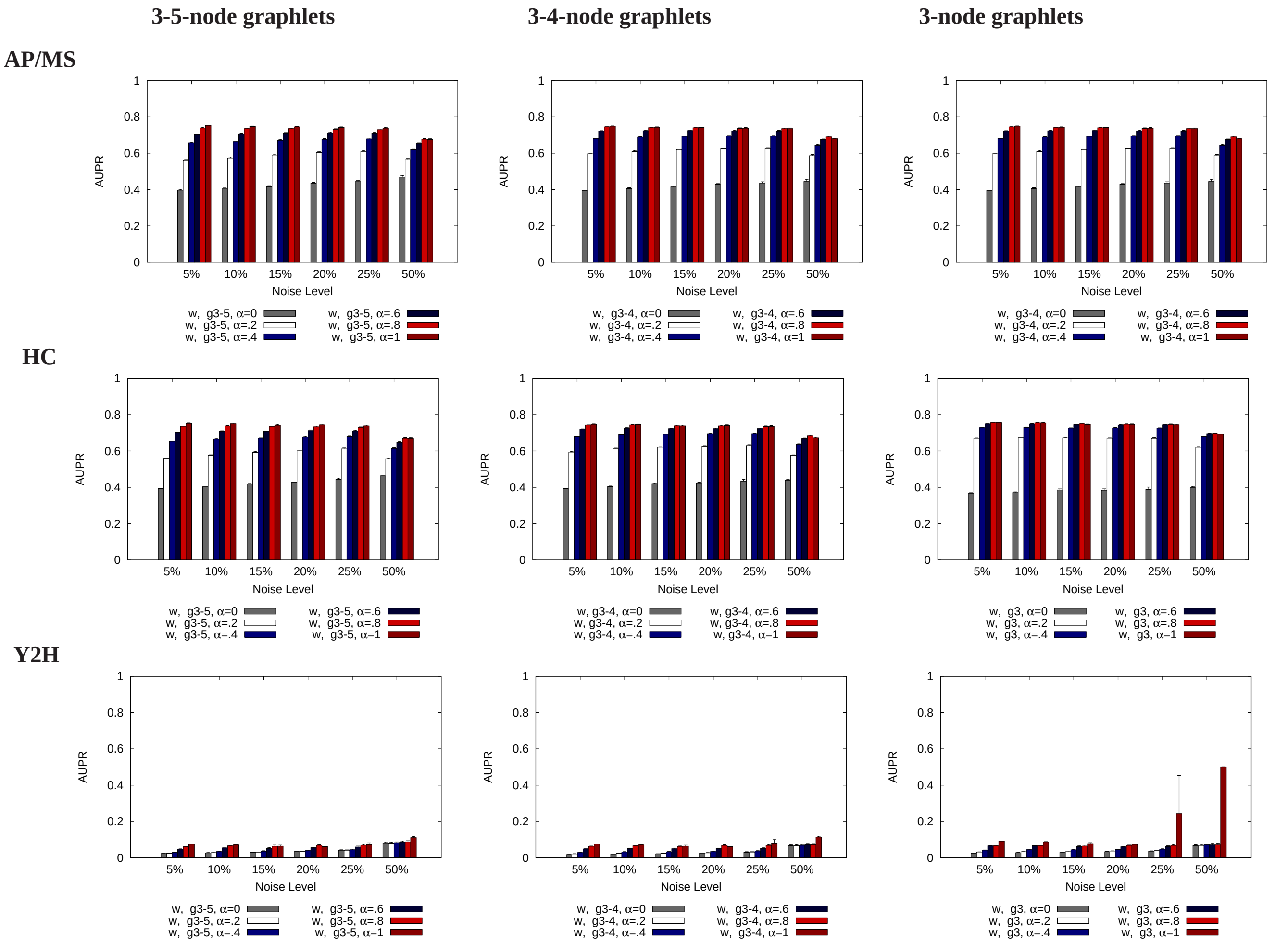

Fig. S10. Link prediction accuracy of the **density-weighted** graphlet-based methods in terms of **AUPRs** at different noise levels as α is varied from 0 to 1. Recall that $\alpha = 0$ corresponds to using node-GDV-similarity alone and $\alpha = 1$ corresponds to using node-pair-GDV-centrality alone. The first column corresponds to using 3-5-node graphlets, the second column corresponds to using 3-4-node graphlets, and the third column corresponds to using 3-node graphlets only. The first row corresponds to the AP/MS network, the second row corresponds to the HC network, and the third row corresponds to the Y2H network. The error bar for a given method corresponds to the standard deviation over five random runs of the method. In some cases, standard deviations are so small that they are not visible.

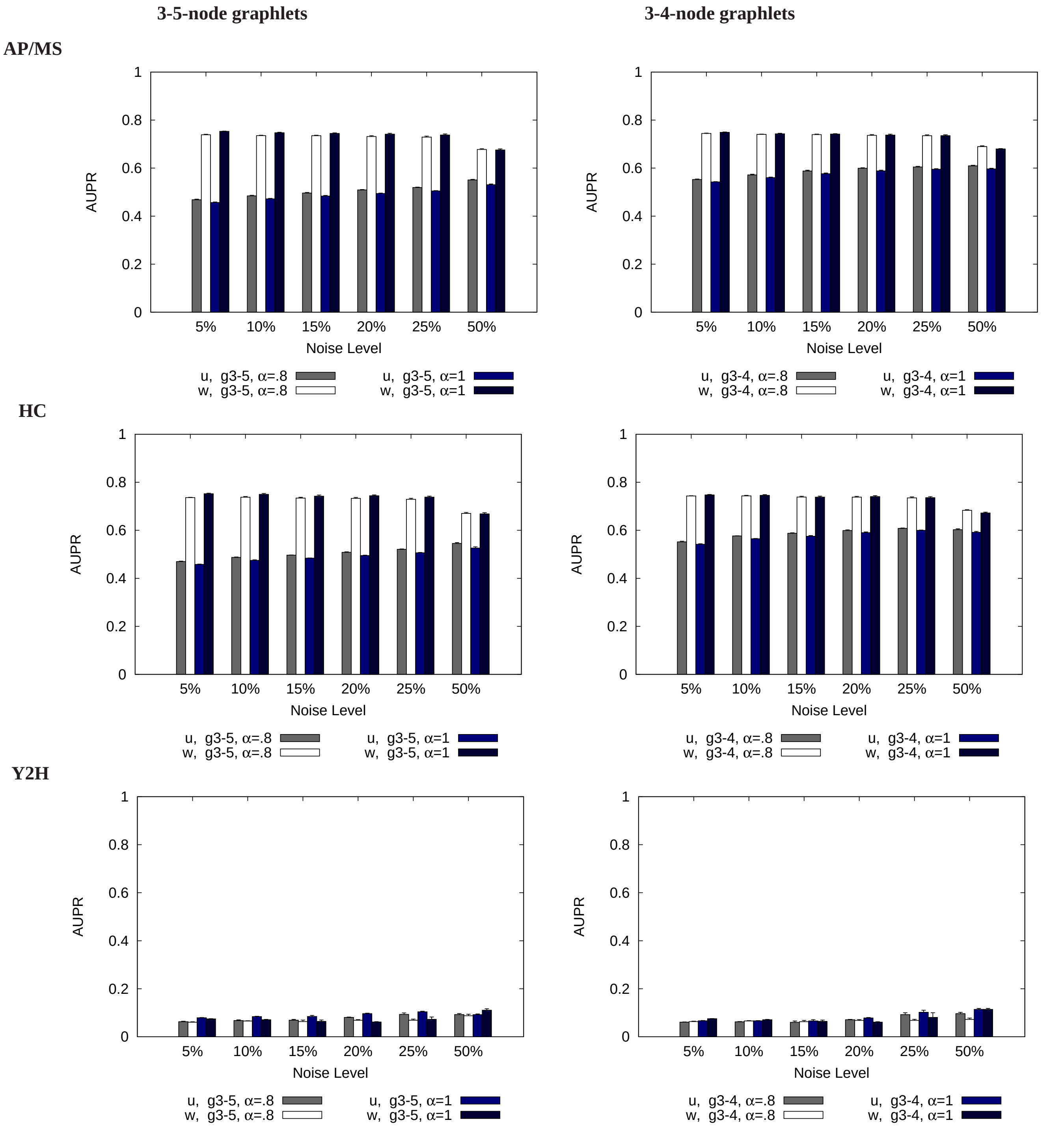

Fig. S11. Link prediction accuracy of the graphlet-based methods in terms of **AUPRs** at different noise levels **comparing unweighted and weighted versions of the methods**. The first column corresponds to using 3-5-node graphlets and the second column corresponds to using 3-4-node graphlets. The results for using 3-node-graphlets are not included, as for this graphlet size, the unweighted and weighted versions of the methods are equivalent. Also, unlike in the following figures, the results for α of 0 are not included in this figure, as at this α, the unweighted and weighted versions of the methods are equivalent. The first row corresponds to the AP/MS network, the second row corresponds to the HC network, and the third row corresponds to the Y2H network. The error bar for a given method corresponds to the standard deviation over five random runs of the method. In some cases, standard deviations are so small that they are not visible.

AP/MS

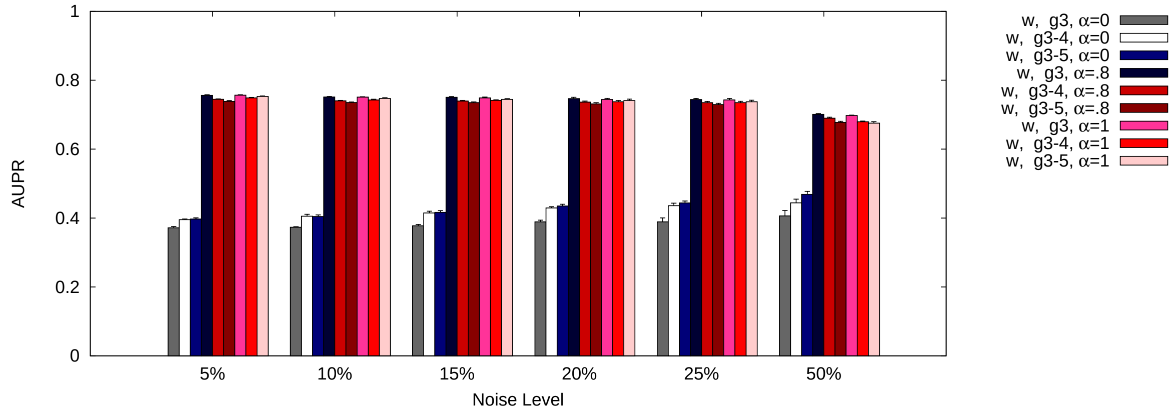

HC

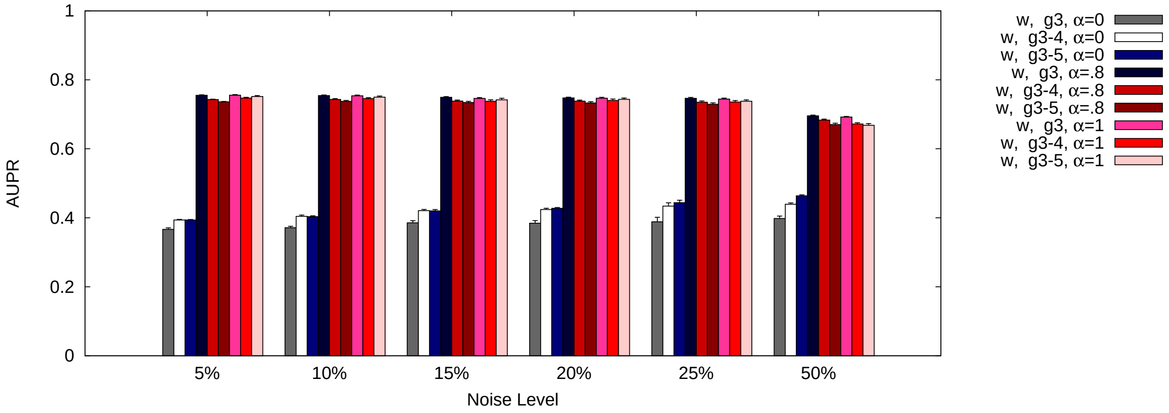

Y2H

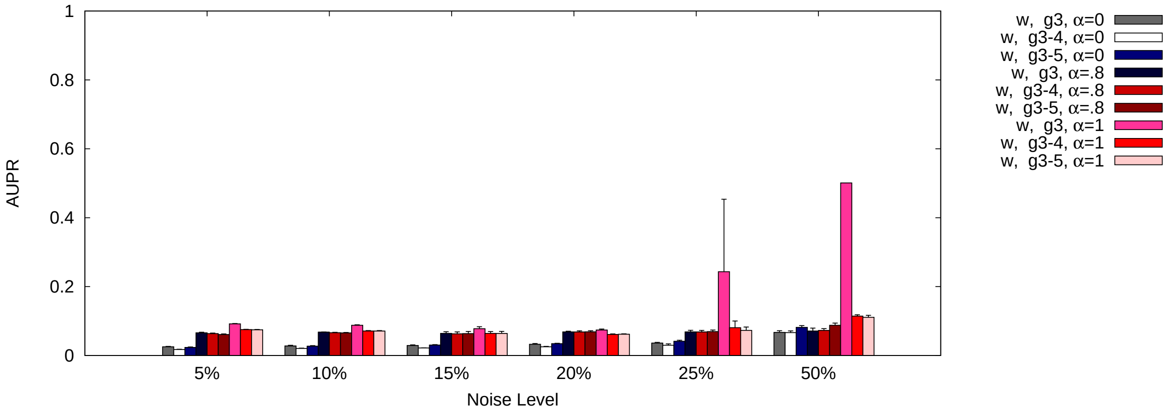

Fig. S12. Link prediction accuracy of the weighted graphlet-based methods in terms of **AUPRs** at different noise levels **comparing different graphlet sizes** in each of the three networks (AP/MS, HC, and Y2H). The error bar for a given method corresponds to the standard deviation over five random runs of the method. In some cases, standard deviations are so small that they are not visible.

AP/MS

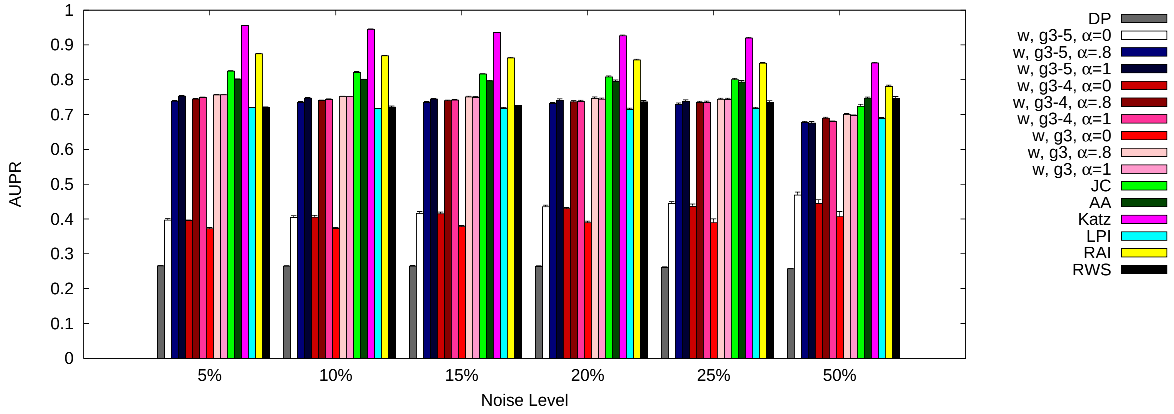

HC

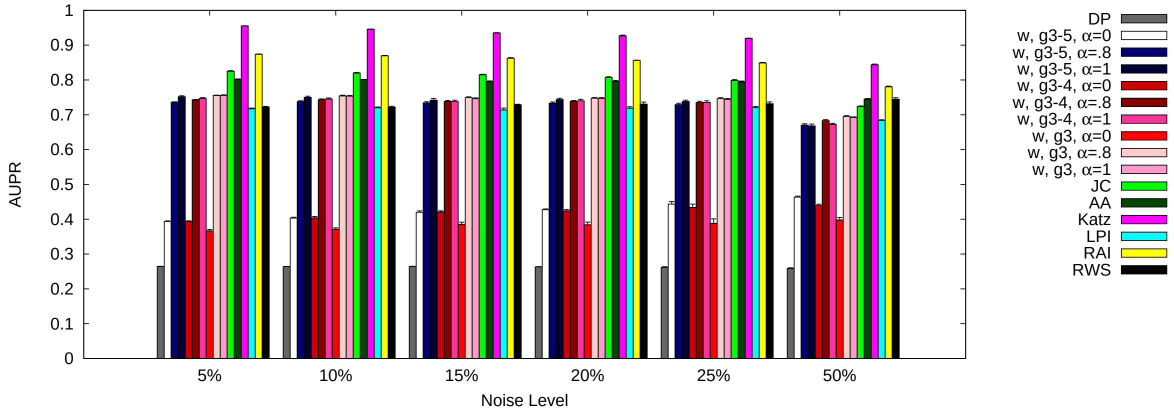

Y2H

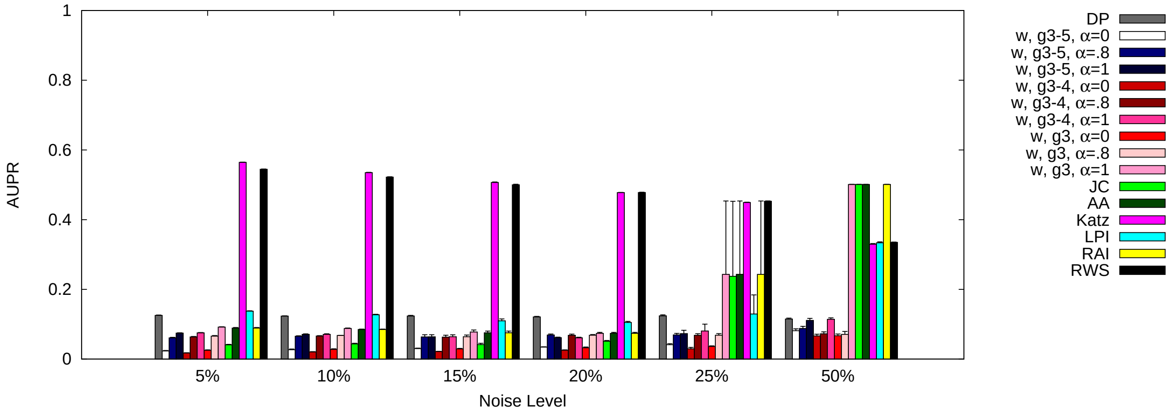

Fig. S13. Link prediction accuracy of our new methods and existing methods in terms of **AUPRs** at different noise levels **comparing the different methods** in each of the three networks (AP/MS, HC, and Y2H). The error bar for a given method corresponds to the standard deviation over five random runs of the method. In some cases, standard deviations are so small that they are not visible.

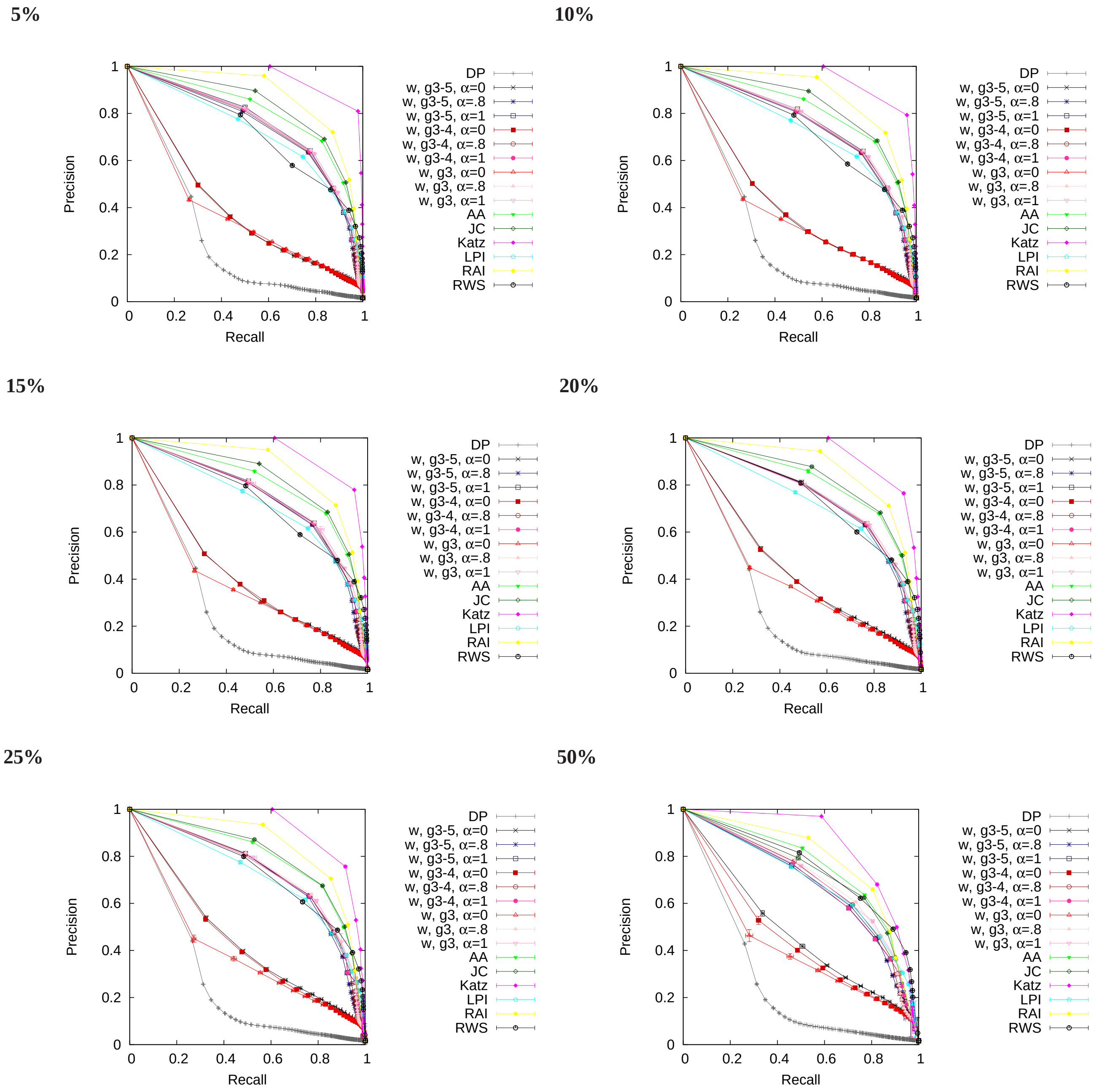

Fig. S14. Precision-recall curves for the AP/MS network for each noise level. The vertical error bar at a given value of k and for a given method corresponds to the standard deviation of precision over five random runs of the method. The horizontal error bar at a given value of k and for a given method corresponds to the standard deviation of recall over five random runs of the method.

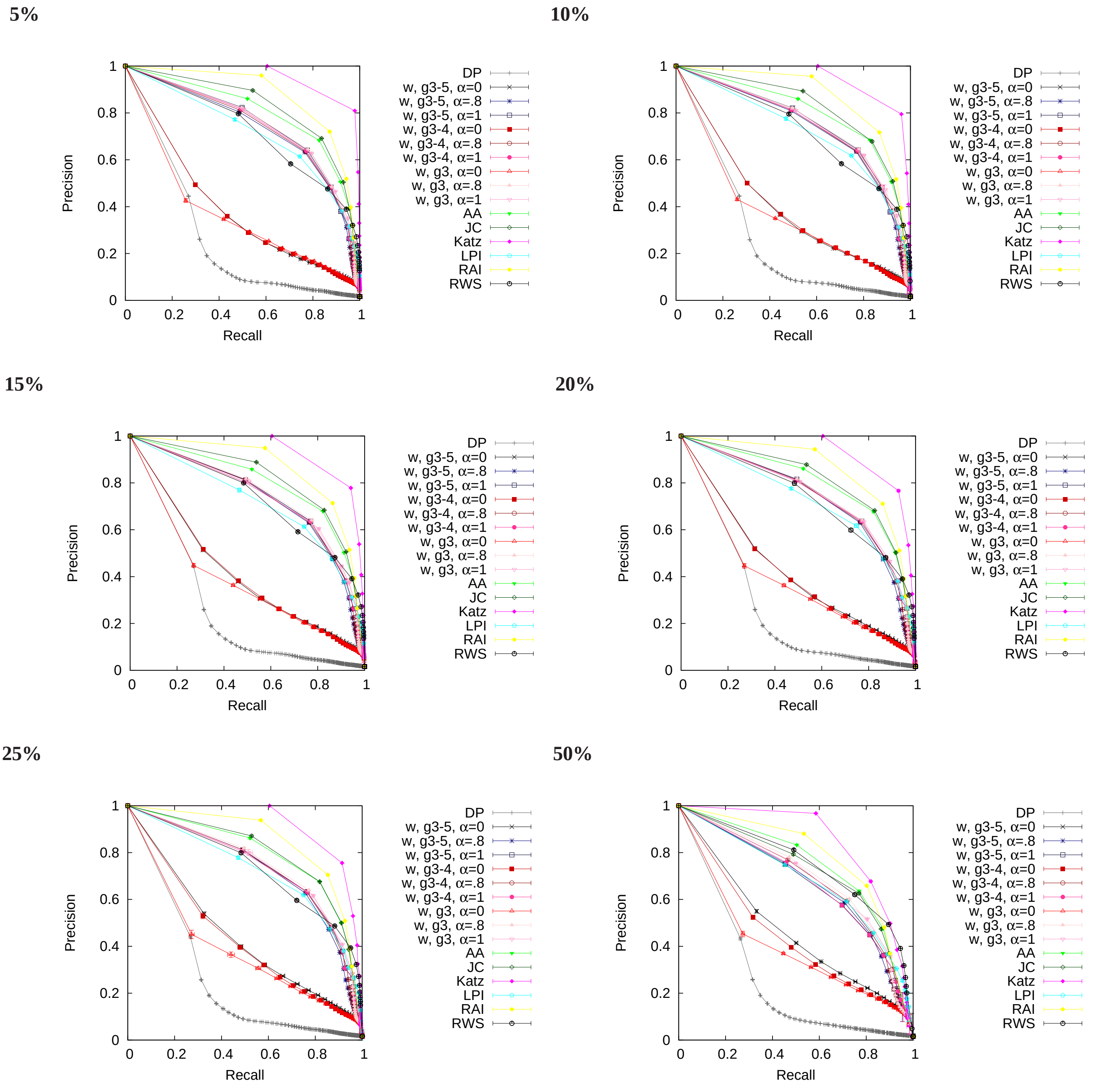

Fig. S15. Precision-recall curves for the HC network for each noise level. The vertical error bar at a given value of k and for a given method corresponds to the standard deviation of precision over five random runs of the method. The horizontal error bar at a given value of k and for a given method corresponds to the standard deviation of recall over five random runs of the method.

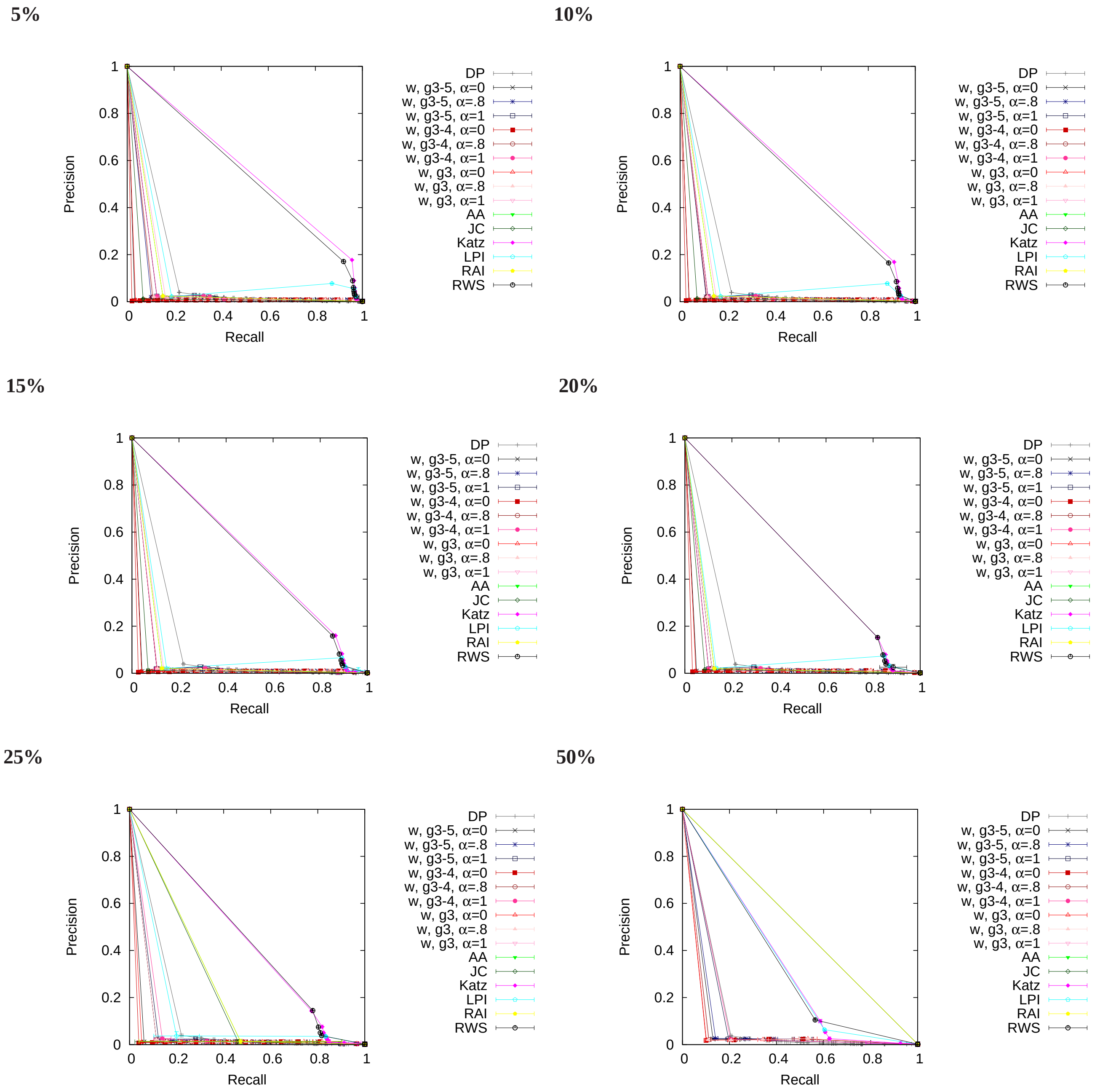

Fig. S16. Precision-recall curves for the Y2H network for each noise level. The vertical error bar at a given value of k and for a given method corresponds to the standard deviation of precision over five random runs of the method. The horizontal error bar at a given value of k and for a given method corresponds to the standard deviation of recall over five random runs of the method.

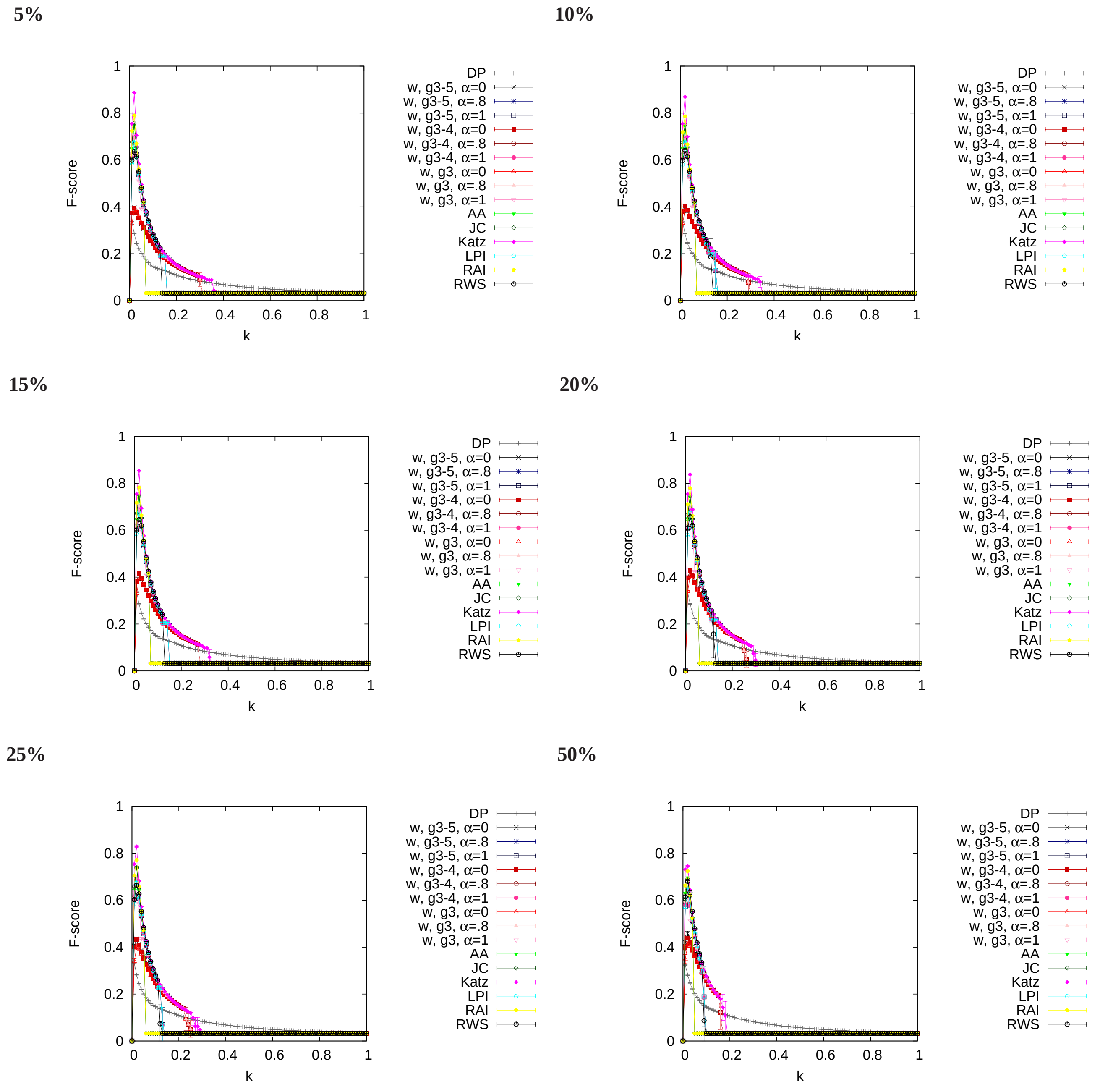

Fig. S17. F-score curves for the AP/MS network for each noise level.

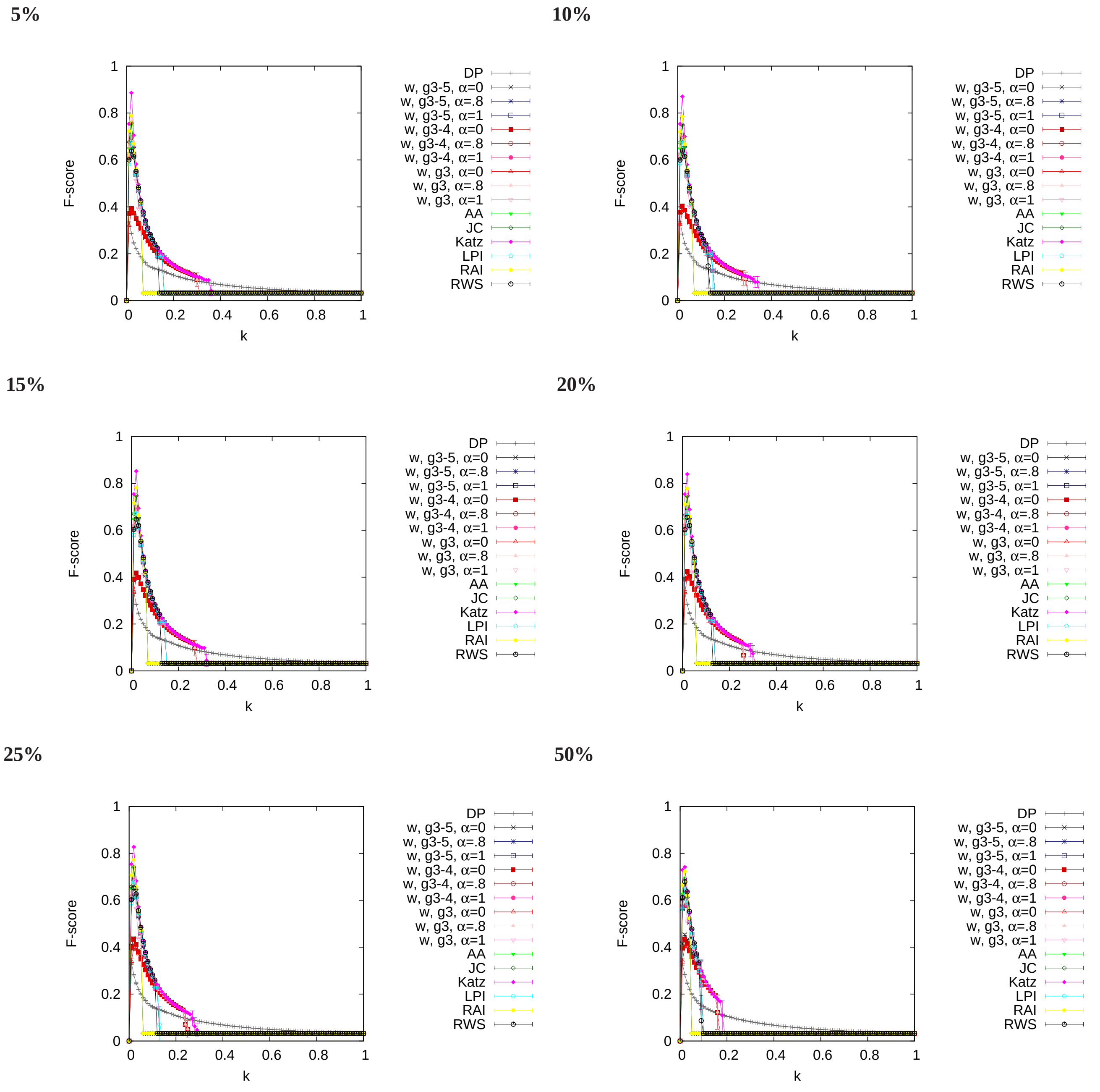

Fig. S18. F-score curves for the HC network for each noise level.

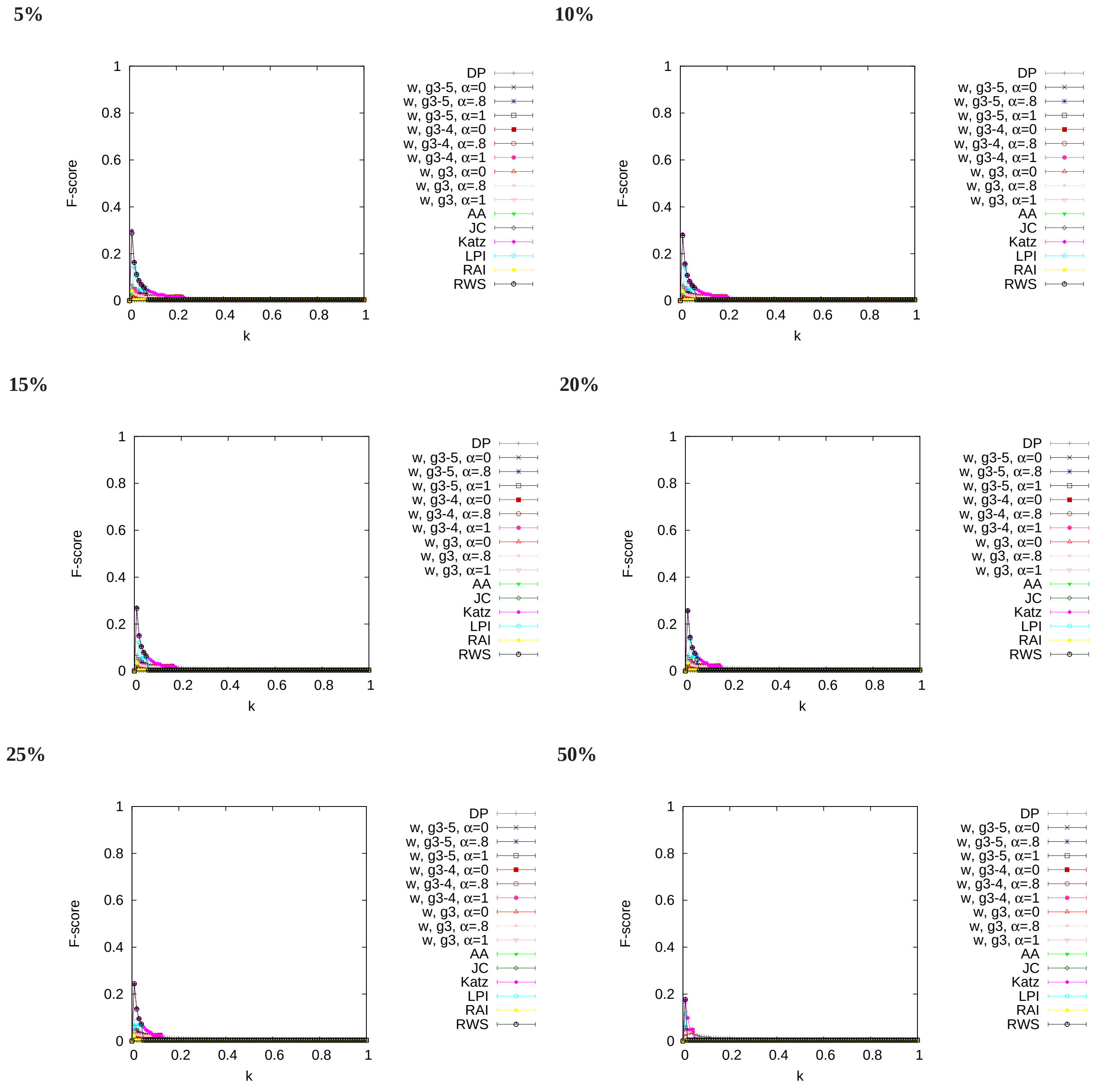

Fig. S19. F-score curves for the Y2H network for each noise level.

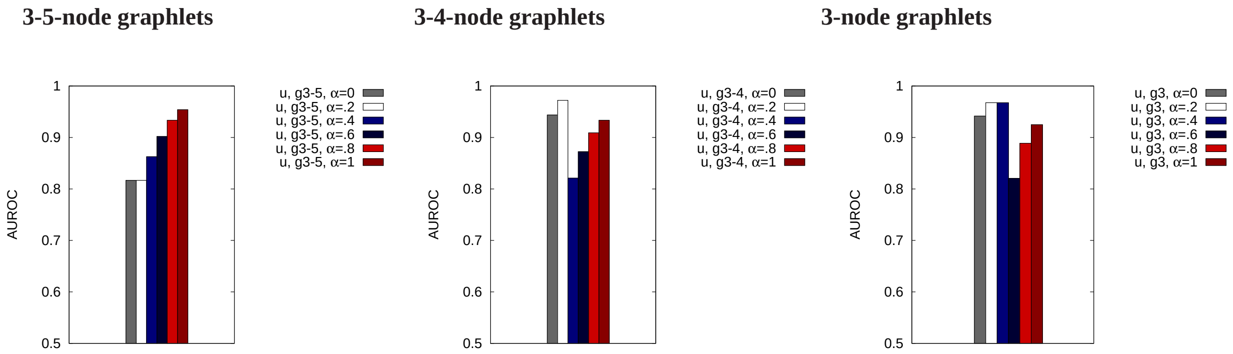

Fig. S20. Link prediction accuracy of the **unweighted** graphlet-based methods in terms of **AUROCs** for the HC network against the low confidence data as α is varied from 0 to 1. Recall that $\alpha = 0$ corresponds to using node-GDV-similarity alone and $\alpha = 1$ corresponds to using node-pair-GDV-centrality alone. The first column corresponds to using 3-5-node graphlets, the second column corresponds to using 3-4-node graphlets, and the third column corresponds to using 3-node graphlets only.

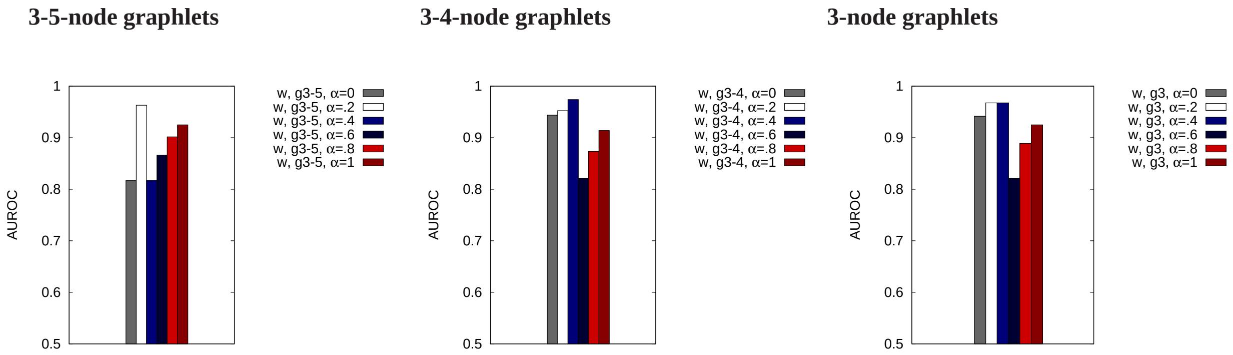

Fig. S21. Link prediction accuracy of the **density-weighted** graphlet-based methods in terms of **AUROCs** for the HC network against the low confidence data as α is varied from 0 to 1. Recall that $\alpha = 0$ corresponds to using node-GDV-similarity alone and $\alpha = 1$ corresponds to using node-pair-GDV-centrality alone. The first column corresponds to using 3-5-node graphlets, the second column corresponds to using 3-4-node graphlets, and the third column corresponds to using 3-node graphlets only.

3-5-node graphlets **3-4-node graphlets**

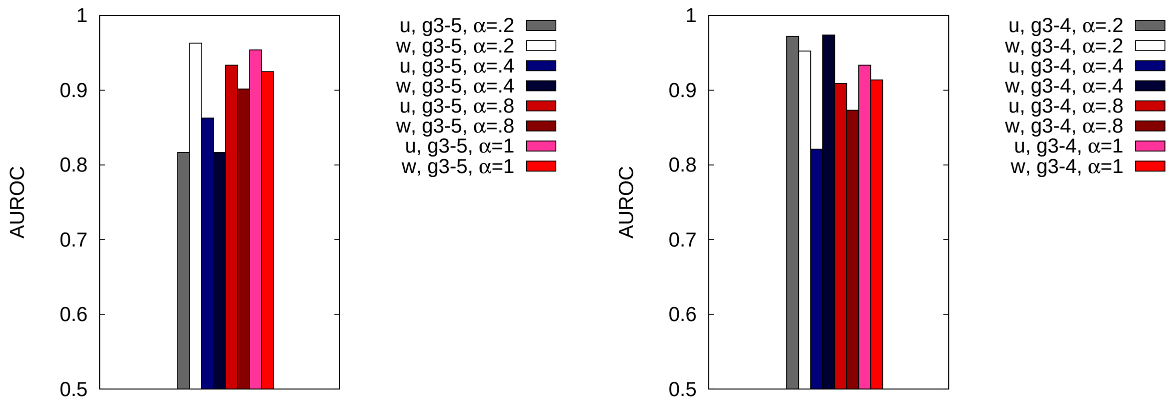

Fig. S22. Link prediction accuracy of the graphlet-based methods in terms of **AUROCs** for the HC network against the low confidence data **comparing unweighted and weighted versions of the methods**. The first column corresponds to using 3-5-node graphlets and the second column corresponds to using 3-4-node graphlets. The results for using 3-node-graphlets are not included, as for this graphlet size, the unweighted and weighted versions of the methods are equivalent. We identify from Figures S20 and S21 the values of α which result in the highest AUROCs, and we include these αs (namely 0.2 and 0.4) into the figure. Also, we include α of 0.8, as this α was the best among all αs when evaluating the methods on noisy networks (Figures S1 and S2). In addition, we include α of 1, as this α corresponds to using only node-pair-GDV-centrality and no node-GDV-similarity in the total link prediction score.

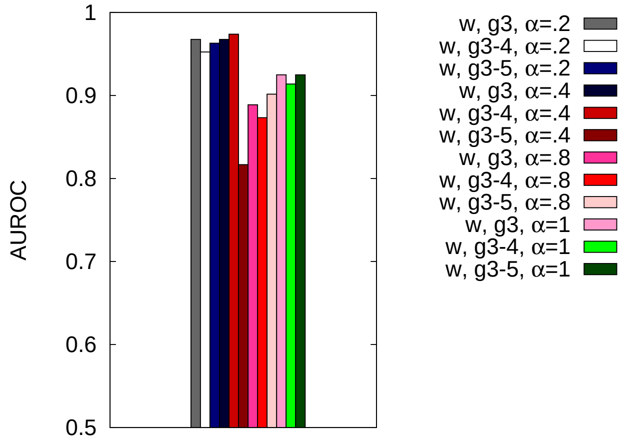

Fig. S23. Link prediction accuracy of the graphlet-based methods in terms of **AUROCs** for the HC network against the low confidence data **comparing different graphlet sizes**. We include into this figure the same αs as in Figure S22.

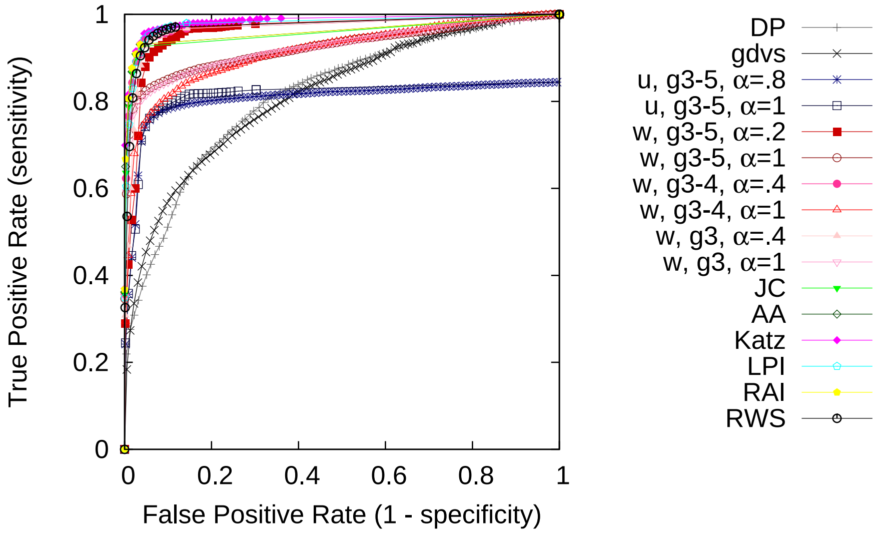

Fig. S24. Receiver operator curves for the HC network against the low confidence data **comparing the different methods**.

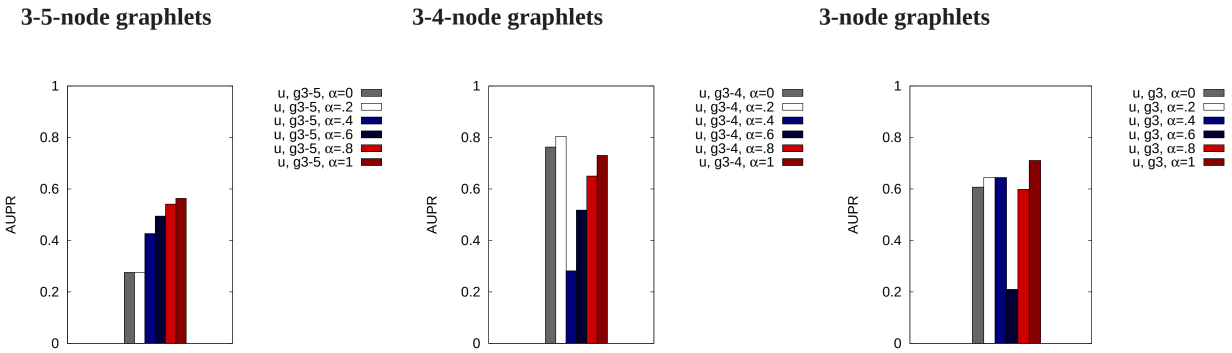

Fig. S25. Link prediction accuracy of the **unweighted** graphlet-based methods in terms of **AUPRs** for the HC network against the low confidence data as α is varied from 0 to 1. Recall that $\alpha = 0$ corresponds to using node-GDV-similarity alone and $\alpha = 1$ corresponds to using node-pair-GDV-centrality alone. The first column corresponds to using 3-5-node graphlets, the second column corresponds to using 3-4-node graphlets, and the third column corresponds to using 3-node graphlets only.

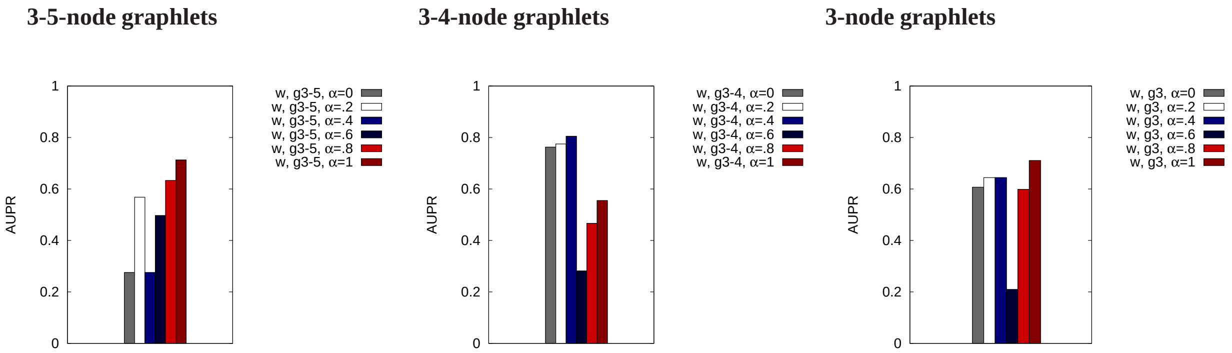

Fig. S26. Link prediction accuracy of the **density-weighted** graphlet-based methods in terms of **AUPRs** for the HC network against the low confidence data as α is varied from 0 to 1. Recall that $\alpha = 0$ corresponds to using node-GDV-similarity alone and $\alpha = 1$ corresponds to using node-pair-GDV-centrality alone. The first column corresponds to using 3-5-node graphlets, the second column corresponds to using 3-4-node graphlets, and the third column corresponds to using 3-node graphlets only.

3-5-node graphlets **3-4-node graphlets**

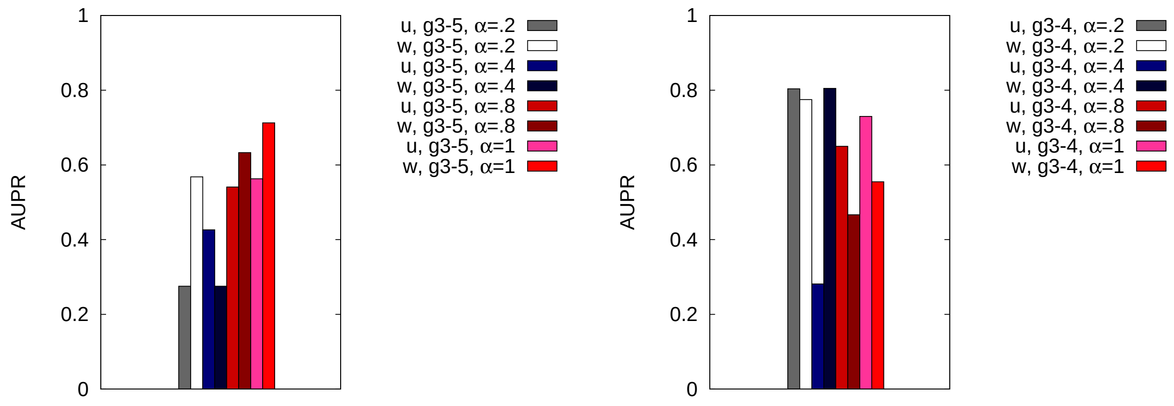

Fig. S27. Link prediction accuracy of the graphlet-based methods in terms of **AUPRs** for the HC network against the low confidence data **comparing unweighted and weighted versions of the methods**. The first column corresponds to using 3-5-node graphlets and the second column corresponds to using 3-4-node graphlets. The results for using 3-node-graphlets are not included, as for this graphlet size, the unweighted and weighted versions of the methods are equivalent. We identify from Figures S20 and S21 the values of α which result in the highest AUPRs, and we include these αs (namely 0.2 and 0.4) into the figure. Also, we include α of 0.8, as this α was the best among all αs when evaluating the methods on noisy networks (Figures S1 and S2). In addition, we include α of 1, as this α corresponds to using only node-pair-GDV-centrality and no node-GDV-similarity in the total link prediction score.

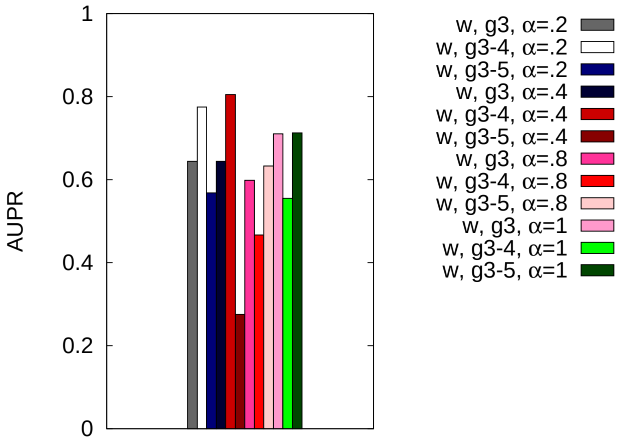

Fig. S28. Link prediction accuracy of the graphlet-based methods in terms of **AUPRs** for the HC network against the low confidence data **comparing different graphlet sizes**. We include into this figure the same αs as in Figure S27.

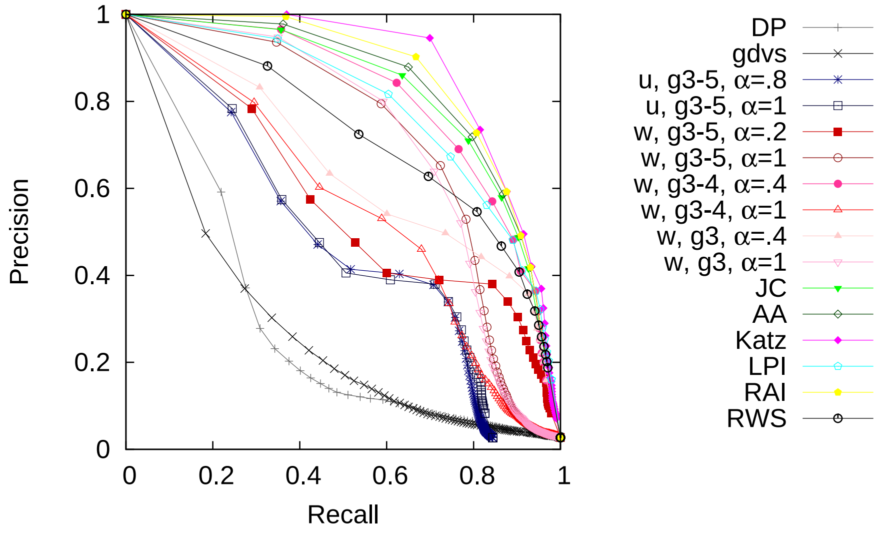

Fig. S29. Precision-recall curves for the HC network against the low confidence data **comparing the different methods**.

AP/MS

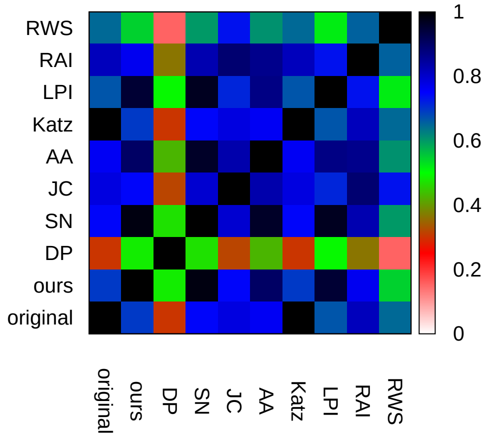

HC

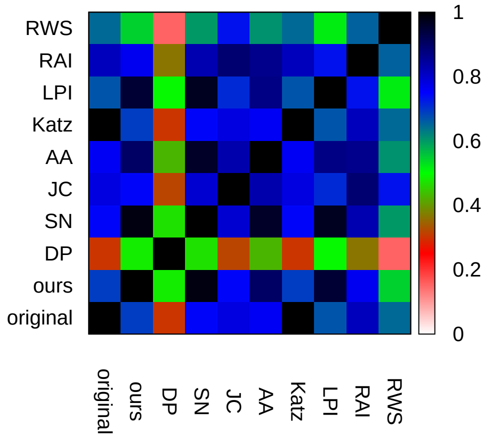

Y2H

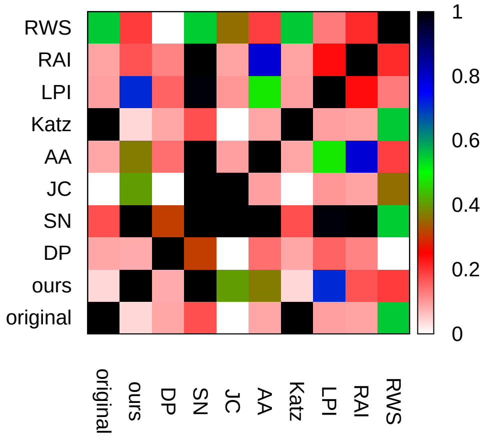

Fig. S30. Pairwise intersections between each pair of de-noised networks as well as between each de-noised network and the original network, for each of the three networks (AP/MS, HC, and Y2H).

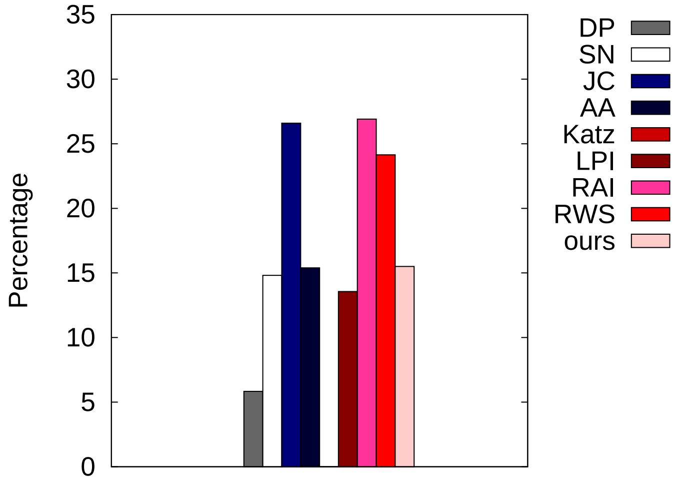

Fig. S31. Percentage of new predicted edges from AP/MS that are validated in BioGRID.
